# Cognitive, perceptual, and motor profiles of school-aged children with developmental coordination disorder

**DOI:** 10.3389/fpsyg.2022.860766

**Published:** 2022-08-03

**Authors:** Dorine Van Dyck, Simon Baijot, Alec Aeby, Xavier De Tiège, Nicolas Deconinck

**Affiliations:** ^1^Laboratoire de Neuroanatomie et Neuroimagerie Translationnelles, ULB Neuroscience Institute, Université libre de Bruxelles, Brussels, Belgium; ^2^Department of Neurology, Hôpital Universitaire des Enfants Reine Fabiola, Université libre de Bruxelles, Brussels, Belgium; ^3^Neuropsychology and Functional Neuroimaging Research Group at Center for Research in Cognition and Neurosciences, ULB Neurosciences Institute, Université libre de Bruxelles, Brussels, Belgium; ^4^Department of Pediatric Neurology, CUB Hôpital Erasme, Hôpital Universitaire de Bruxelles, Université libre de Bruxelles, Brussels, Belgium; ^5^Department of Translational Neuroimaging, CUB Hôpital Erasme, Hôpital Universitaire de Bruxelles, Université libre de Bruxelles, Brussels, Belgium

**Keywords:** Developmental coordination disorder, subtypes, cluster analysis, visual perceptual skills, executive functions

## Abstract

Developmental coordination disorder (DCD) is a heterogeneous condition. Besides motor impairments, children with DCD often exhibit poor visual perceptual skills and executive functions. This study aimed to characterize the motor, perceptual, and cognitive profiles of children with DCD at the group level and in terms of subtypes. A total of 50 children with DCD and 31 typically developing (TD) peers (7–11 years old) underwent a comprehensive neuropsychological (15 tests) and motor (three subscales of the Movement Assessment Battery for Children-2) assessment. The percentage of children with DCD showing impairments in each measurement was first described. Hierarchical agglomerative and K-means iterative partitioning clustering analyses were then performed to distinguish the subtypes present among the complete sample of children (DCD and TD) in a data-driven way. Moderate to large percentages of children with DCD showed impaired executive functions (92%) and praxis (meaningless gestures and postures, 68%), as well as attentional (52%), visual perceptual (46%), and visuomotor (36%) skills. Clustering analyses identified five subtypes, four of them mainly consisting of children with DCD and one of TD children. These subtypes were characterized by: (i) generalized impairments (8 children with DCD), (ii) impaired manual dexterity, poor balance (static/dynamic), planning, and alertness (15 DCD and 1 TD child), (iii) impaired manual dexterity, cognitive inhibition, and poor visual perception (11 children with DCD), (iv) impaired manual dexterity and cognitive inhibition (15 DCD and 5 TD children), and (v) no impairment (25 TD and 1 child with DCD). Besides subtle differences, the motor and praxis measures did not enable to discriminate between the four subtypes of children with DCD. The subtypes were, however, characterized by distinct perceptual or cognitive impairments. These results highlight the importance of assessing exhaustively the perceptual and cognitive skills of children with DCD.

## Introduction

Children with developmental coordination disorder (DCD) have fine and/or gross motor skills below the level expected for their age and learning opportunities. According to the DSM-5 criteria (American Psychiatric Association [APA], 2013), these motor impairments are not better accounted for by any other medical, neurodevelopmental, psychological, social condition, or cultural background, occur in the early developmental period, and interfere with several areas of daily living (i.e., school or work productivity, home life, play and leisure activities). In DCD, co-occurring conditions are the rule rather than the exception (Kaplan et al., 1998; Visser, 2003). One of the most common co-occurring conditions is attention-deficit/hyperactivity disorder (ADHD), which is found in up to 50% of DCD cases (Kadesjö and Gillberg, 1998). ADHD is a neurodevelopmental disorder characterized by attention impairments, impulsivity, or hyperactivity (American Psychiatric Association [APA], 2013).

DCD has been described as a collection of conditions (Vaivre-Douret, 2014), given its high clinical heterogeneity (Visser, 2003). Children with DCD display numerous clinical motor manifestations, varying greatly in nature and severity within and between individuals. For instance, children with DCD might have limitations in sports performance, ball skills, daily activities such as dressing or using cutlery, handwriting, and/or fine manipulation. Thus, DCD may affect either all motor skills, some motor skills, or some motor skills more than others (Miller et al., 2001; Magalhães et al., 2011). DCD is also much more than a motor disorder (e.g., Wilson et al., 2013; Zwicker et al., 2018), since the literature reports frequent visual perceptual (Wilson and McKenzie, 1998; Schoemaker et al., 2001; Van Waelvelde et al., 2004; Tsai et al., 2008; Cheng et al., 2014) and cognitive impairments, such as reduced executive functions (i.e., enabling behavioral adjustment when automatic processes are not sufficient; Diamond, 2013; Wilson et al., 2013, 2017) or short-term memory (e.g., Alloway, 2011).

Visual perceptual impairments have frequently been reported in DCD, whether the task involves a motor component or not (Wilson and McKenzie, 1998; Schoemaker et al., 2001; Van Waelvelde et al., 2004; Tsai et al., 2008; Wilson et al., 2013; Cheng et al., 2014; Prunty et al., 2016; Micheletti et al., 2021). These perceptual impairments include visual and visuospatial skills, such as basic form detection, visual discrimination, motion detection, and visuospatial processing. Moreover, cognitive functions based on visual and visuospatial skills are also impaired, namely visual and visuospatial short-term or working memory (Tsai et al., 2008; Alloway, 2011; Cheng et al., 2014). Yet, poor performance is not generalized across the entire DCD population (Hoare, 1994; Schoemaker et al., 2001; Van Waelvelde et al., 2004; Tsai et al., 2008) or across all tasks (Schoemaker et al., 2001; Tsai et al., 2008; Prunty et al., 2016). Regarding individual performance, 5–47% of children with DCD were impaired in visual or visuospatial perceptual measures, and 5–42% in visuomotor measures (Schoemaker et al., 2001; Tsai et al., 2008). Identifying visual perceptual impairments is particularly important as these might lead to motor coordination impairments, and impact the selection of the most appropriate intervention plan (e.g., Chokron and Dutton, 2016). Visuospatial and visuomotor skills are also associated with academic (i.e., reading, written expression, and mathematics) performance (Carlson et al., 2013; Hopkins et al., 2019), and reduced visuomotor integration might lead to poor spatial organization of written language and mathematics (Barnhardt et al., 2005). It is noteworthy that children with DCD are also at higher risk of having ophthalmic or orthoptic abnormalities, such as abnormal binocular vision (i.e., stereopsis and fusion) and refractive errors (i.e., hypermetropia, myopia, and anisometropia; Creavin et al., 2014). However, the association with perceptual skills has not yet been addressed.

In addition to motor and perceptual impairments, poor executive functions are found in 40–60% of DCD cases (Wilson et al., 2017, 2020). More specifically, children with DCD might have reduced working memory (Alloway, 2011; Leonard et al., 2015), motor (Leonard et al., 2015) and/or cognitive (Pratt et al., 2014; Bernardi et al., 2016) inhibition, as well as difficulties in planning (Pratt et al., 2014) or shifting (i.e., mental flexibility; Wuang et al., 2011). The identification of such impairments appears critical, as executive functions are highly associated with academic performance (e.g., Best et al., 2011; Gerst et al., 2017), social development (e.g., van Lier and Deater-Deckard, 2016), as well as behavioral and emotional regulation (e.g., Ardila, 2013; Predescu et al., 2020). Furthermore, the risk of displaying persistent motor difficulties is higher in children with DCD and low executive functions than in their peers with preserved executive functions (Wilson et al., 2020). These impairments cannot only be accounted for by co-occurring disorders known to be associated with reduced executive functions such as ADHD, as most of the studies mentioned co-occurring ADHD diagnosis as an exclusion criterion (e.g., Pratt et al., 2014; Leonard et al., 2015; Wilson et al., 2020).

To better understand the heterogeneity of DCD and propose specialized interventions, several studies attempted to classify DCD into subtypes based on their clinical manifestations (see Supplementary Material 1 for details; Dewey and Kaplan, 1994; Hoare, 1994; Wright and Sugden, 1996; Macnab et al., 2001; Tsai et al., 2008; Vaivre-Douret et al., 2011; along with learning disabilities: Miyahara, 1994; Pieters et al., 2015). To this end, a statistical approach (i.e., clustering methods) was used to mathematically define subtypes (i.e., clusters) based on their similarities and differences across several clinical variables measuring few dimensions of interest (Hoare, 1994). Some studies focused mainly on the motor skills of children with diagnosed or at risk of having DCD (Miyahara, 1994; Wright and Sugden, 1996), while others included visual perceptual skills (Hoare, 1994; Macnab et al., 2001; Tsai et al., 2008) or transitive gestures (Dewey and Kaplan, 1994), thereby resulting in discrepancies in the number and characteristics of the clusters.

Hoare (1994) found five subtypes in a sample of 80 children having DCD (aged 6–9 years old [y.o.]), characterized by: (i) below average running and kinesthetic acuity, along with above average manual dexterity and static balance, (ii) above average visual perceptual/visuomotor skills, (iii) generalized perceptuo-motor impairments (except for running, around average), (iv) below average visual perceptual skills, with above average manual dexterity, kinesthetic acuity, and running, (v) motor execution problems (i.e., below average manual dexterity, static balance and running, with above average kinesthetic acuity). These results highlighted that a subtype of children with DCD might suffer from severe visual perceptual impairments, and that there were dissociations between perceptual skills (visual perceptual and kinesthetic skills). When adding a sample of 20 typically developing (TD) children, a new cluster was created, comprised of most of the TD children (*n* = 18) and 8 children with DCD. The use of distinct variables measuring similar underlying dimensions in another sample of 62 children with DCD (aged 7–12 y.o.) reproduced the general structure of the subtypes, yet with variations in the proportion of children within each subtype (Macnab et al., 2001). Dewey and Kaplan (1994) found four subtypes among a sample of 51 children at risk of having DCD (i.e., referred by school teachers for motor problems, and scoring below –1 standard deviation on one motor screening test) and 51 TD children (aged 6–10 y.o.), characterized by: (i) generalized impairments, (ii) reduced balance, coordination (upper limbs and bilateral), and gestural performance, (iii) reduced motor sequencing, and (iv) no impairment (12 children at risk of having DCD and 50 TD children). These results show the presence of dissociations in DCD (at risk) population between deficits in motor planning and execution, as the second subtype had reduced performance on tests assessing motor execution and intact motor planning (i.e., motor sequencing), while the opposite was the case for the third subtype. Based on the Movement Assessment Battery for Children (M-ABC) assessment and checklist, Wright and Sugden (1996) found four subtypes in a sample of 69 children at risk of having DCD (i.e., scoring below the 5th or the 15th percentile on the M-ABC checklist; aged 6–9 y.o.): (i) mild impairments (i.e., scores around or above the average of the whole group), (ii) below average catching, with above average control of self (i.e., respect their own timing in a stable environment), (iii) generalized impairments, (iv) below average skills to move their hands quickly, dynamic balance, and adapt to changing environment, with above average catching. These results stressed the existence of dissociations between and within fine and gross motor performance in children at risk of having DCD. For instance, the second subtype had below average catching, along with around or above average fine motor skills and other gross motor skills, while the opposite was seen for the fourth subtype. More recently, Tsai et al. (2008) identified four subtypes in a sample of 178 children at risk of having DCD (i.e., scoring below the 5th percentile on the M-ABC; 9–10 y.o.), characterized by: (i) below average (relative to peers at risk of having DCD) manual dexterity, static balance, and visual perceptual skills, with above average ball skills, (ii) below average manual dexterity (except for one subtest), ball skills, and static balance, with above average visual perceptual skills and dynamic balance, (iii) below average ball skills, with above average visual perceptual skills, manual dexterity (except for one subtest), static and dynamic balance, (iv) generalized impairments (except for ball skills). These results emphasize the presence of dissociations within fine and gross motor skills, and suggest that visual perceptual impairments are not specific to one subtype (Tsai et al., 2008).

More recently, four research groups included a larger number of cognitive measures (Vaivre-Douret et al., 2011; Lalanne et al., 2012; Asonitou and Koutsouki, 2016; Costini et al., 2017; Lust et al., 2022). Vaivre-Douret et al. (2011) first used inferential clinical analysis to classify their sample of 43 children with DCD (aged 5–15 y.o.) into three subtypes. These subtypes were reproduced by clustering analysis (misclassification of four children) performed on neuropsychological (e.g., intellectual functions, visual perceptual, visuospatial, visuomotor, and visual constructional skills, executive functions, language), neuropsychomotor (e.g., manual dexterity, praxis, digital gnosis, rhythmic skills, gross motor-control skills, etc.) and neurovisual (i.e., electro-oculogram and electro-retinogram) assessments. One subtype (“ideomotor”) had impairments in motor programming and planning, postural control (i.e., balance, hypotonia), and preserved visual perceptual skills. The second subtype (“visuospatial and visual constructional”) had impaired visual perceptual and visuospatial skills, while the last subtype (“mixed”) showed generalized impairments. Later, using multivariate analyses on the same (but extended; *n* = 63) sample, the same research group (Lalanne et al., 2012) confirmed that measures assessing motor programming and planning (i.e., imitation of meaningless gestures and digital praxis) discriminated the first subtype, and visuomotor integration and visuospatial structuring the second subtype. In addition to having characteristics of the first two subtypes, the “mixed” subtype was also characterized by coordination difficulties (i.e., upper-lower limbs coordination, manual dexterity, and synkinesia). The assessment method selected by the authors of the two studies to assess motor skills (“neuropsychomotor functions in children,” NP-MOT; Vaivre-Douret, 2006) is only standardized until the age of 8 years. This choice may have had an impact on the proportion of children belonging to the first subtype. It might also have led to subtle differences compared with previous studies dealing with DCD subtypes in terms of sample recruited and performances of the subtypes. In the second study, Asonitou and Koutsouki (2016) identified six subtypes in a sample of 54 preschool (5–6 y.o.) children identified with DCD based on the M-ABC score and parents’ reports, and 54 TD peers. Five subtypes included children with DCD (three of them—i, ii, v—also comprised TD children), characterized by: (i) reduced jumping, and minor difficulties with manual dexterity and simultaneous coding, (ii) reduced manual dexterity, planning, and simultaneous coding, (iii) reduced manual dexterity, static/dynamic balance, and planning, (iv) generalized impairment, and (v) no impairment. A sixth subtype was composed exclusively of TD children. Their results showed that reduced attention and executive functions were present in all subtypes of children with DCD rather than in one specific subtype and were highly associated with motor impairments in preschool children. In a multiple case study, Costini et al. (2017) classified 27 children with DCD (aged 7–13 y.o.) into four subtypes employing a procedure of progressive inclusion. They demonstrated that DCD is rarely purely motor. The first three subtypes were characterized by deficits in: (i) visual perceptual and visuospatial skills, (ii) executive functions, and (iii) gestural conceptual knowledge. The fourth subtype, encompassing nine children, did not show any of the above-mentioned impairments. This subtype remained heterogeneous, as the children had no common characteristics, and was thus labeled “others” by the authors. Lastly, Lust et al. (2022) investigated motor, visual perceptual and visuomotor skills along with intellectual functions in 98 children with DCD. Their clustering analysis found two subtypes characterized by various degrees of generalized below or around average performance (relative to peers with DCD), one subtype with above average performance, except for gross motor skills (i.e., ball skills and balance; MABC-2), and one subtype characterized by below average performance in fine motor skills, visual perceptual skills, and perceptual reasoning. Their results showed that a large percentage of children (56%) have generalized rather than specific motor, perceptual and cognitive impairments. Moreover, reduced perceptual reasoning contrasting with averaged verbal skills is specific to a subtype of children whose profile is also characterized by poor fine motor and visual perceptual skills.

Although no clear consensus has emerged from the existing literature, all studies based on clustering analyses identified a subtype of children characterized by generalized impairments (i.e., impairment or poorer performance than TD or DCD peers in most of the measures selected; Dewey and Kaplan, 1994; Hoare, 1994; Wright and Sugden, 1996; Macnab et al., 2001; Tsai et al., 2008; Vaivre-Douret et al., 2011; Asonitou and Koutsouki, 2016). In addition, the authors who included children with DCD and TD peers in their clustering analysis found one mixed subtype comprising both children with DCD and mild impairments, and TD children (Dewey and Kaplan, 1994; Hoare, 1994; Asonitou and Koutsouki, 2016).

Most of these previous studies focused either exclusively on motor measures or on motor measures along with another major dimension impaired in DCD (e.g., visual perceptual skills or executive functions) in order to classify their DCD samples into subtypes. However, a comprehensive assessment of motor skills, visual perceptual skills, and cognitive functions while also defining subtypes would enable to best capture the overall functioning of children with DCD. Characterizing these subtypes would help to explain discrepancies in the literature, and to better understand the heterogeneity and underlying mechanisms of the disorder by performing subtype comparisons. Accordingly, the clinical manifestations of the different subtypes might be underpinned by specific neural bases and explained by impairments in distinct cognitive or motor processes. Considering the intra-group variability of DCD will also help determine whether the subtypes of children with DCD differ in nature (i.e., qualitative difference) and/or severity (i.e., quantitative difference). The approach of neurodevelopmental disorders that was initially categorical tends to evolve toward a dimensional or continuous approach (Peters and Ansari, 2019). According to the latter, neurodevelopmental disorders are at the end of a continuum along the normal distribution (e.g., in ADHD: Balázs and Keresztény, 2014; Drechsler et al., 2020; in autism spectrum disorder—ASD: Constantino and Todd, 2003; Skuse et al., 2009; in reading and arithmetic disorders: Snowling and Hulme, 2012; Peters and Ansari, 2019). Finally, subtype characterization would also help to propose specialized interventions based on the main impairments of the different subtypes.

This study was aimed at characterizing the motor, perceptual, and cognitive profiles of school-aged children with DCD at the group level and in terms of subtypes. For that purpose, a large group of children with DCD and age-matched TD peers underwent comprehensive motor (i.e., fine and gross motor skills) and neuropsychological (i.e., executive and attentional functions, short-term memory, visual perceptual and visuomotor skills, praxis, intellectual functions) assessments. Firstly, we described the percentage of children with impairments in each of these main functional areas. We expected to find a certain percentage of children among the sample of children with DCD with impaired executive functions (e.g., Leonard et al., 2015; Wilson et al., 2020) and impaired visual perceptual skills, whether involving a motor component or not (e.g., Schoemaker et al., 2001; Tsai et al., 2008). Secondly, we performed statistical clustering analyses to classify the complete sample of children (DCD and TD) into subtypes based on their motor, perceptual, and cognitive performances. We expected to identify several subtypes based on the nature (i.e., motor, perceptual, and cognitive) and severity (i.e., generalized impairments or mild impairments subtypes) of the children’s difficulties. More precisely, we expected to find at least four clusters, characterized by: (i) severe generalized impairments, (ii) mainly visual and visuospatial perceptual impairments, (iii) motor impairments and preserved perceptual skills, and (iv) no impairment (mainly composed of TD children; Dewey and Kaplan, 1994; Hoare, 1994; Wright and Sugden, 1996; Macnab et al., 2001; Tsai et al., 2008; Vaivre-Douret et al., 2011; Asonitou and Koutsouki, 2016). Moreover, we assumed that reduced executive functions and attention would be present across all the clusters with impairments (Asonitou and Koutsouki, 2016). Co-occurring ADHD was not an exclusion criteria given its high prevalence in this population and the investigation of executive functions (Kadesjö and Gillberg, 1998). TD children were included in the clustering analyses to validate this statistical approach by the classification into a cluster with no impairments.

## Materials and methods

### Participants

A total of 103 children, aged 7–11 y.o., were enrolled in the study, with 63 assigned to the DCD group and 40 to the TD group. Children with a diagnosis confirmed by a pediatric neurologist or a suspected DCD (i.e., parents’ or schools’ concerns regarding the child’s motor skills leading to a clinical consultation) were recruited. In the case of a suspected DCD, the standardized clinical assessment became part of the diagnostic process and enabled confirming or ruling out the diagnosis of DCD. Overall, 18 children met one of the exclusion criteria, as described below (DCD: *n* = 10; TD: *n* = 8), and four children dropped out of the study (DCD: *n* = 3; TD: *n* = 1). After the exclusion of these 22 children, the final sample was comprised of 50 children with DCD (8 females and 42 males; mean ± *SD* age: 9.51 ± 1.54) and 31 TD children (12 females and 19 males; mean ± *SD* age: 9.86 ± 1.40). Descriptive demographic and clinical data from both groups are presented in Table 1.

**TABLE 1:** Demographic and clinical characteristics of the two samples: children with developmental coordination disorder (DCD) and with typical development (TD).

	DCD (*n* = 50)	TD (*n* = 31)	Statistics	*p*
***Demographics and clinical characteristics***				
Sex, *n F/M*	8/42	12/19	X^2^(1) = 5.31	0.02**
Laterality, *n R/L/A*	41/7/2	29/2/0	X^2^(2) = 2.52	0.28
Age (years)	9.51 ± 1.54	9.86 ± 1.40	*U* = 677^a^	0.34
Socioeconomic status	8.73 ± 2.46	10.97 ± 1.99	*U* = 308^a^	<0.001**
***Questionnaires***				
DCD-Q	36.92 ± 11.42	65.28 ± 6.24	*t* (75.6) = –14.17^b^	<0.001**
ADHD-RS-IV	29.36 ± 11.78	11.41 ± 7.66	*t* (75.9) = 8.20^b^	<0.001**
Pathological ADHD-RS-IV score, *yes/no*	28/22	0/31	–	–
***Fine and gross motor skills***				
MABC-2 (percentile)	3.07 ± 3.73	51.13 ± 21.75	*U* = 0^a^	<0.001**
Manual dexterity (standard score)	3.60 ± 1.46	9.39 ± 2.58	*U* = 38^a^	<0.001**
Aiming and catching (standard score)	6.94 ± 2.38	10.10 ± 2.43	*U* = 279^a^	<0.001**
Static and dynamic balance (standard score)	5.58 ± 2.89	11.32 ± 1.40	*t* (75.5) = –11.98^b^	<0.001**
***Neuropsychological assessment***				
Intellectual functions (verbal comprehension index)	103.40 ± 14.48	115.65 ± 12.08	*t* (79) = –3.93	<0.001**
Meaningless postures (imitating hand positions)	17.12 ± 3.83	21.87 ± 2.29	*U* = 226^a^	<0.001**
Meaningless gestures (manual motor sequences)	36.76 ± 7.24	47.84 ± 5.59	*t* (79) = –7.28	<0.001**
Visual perception (visual closure)	9.56 ± 5.81	17.06 ± 3.05	*U* = 220.5^a^	<0.001**
Visuospatial perception (directional relations)	7.82 ± 4.99	3.55 ± 3.27	U = 376^a^	<0.001**
Eye-hand coordination	148.82 ± 20.72	174.06 ± 6.57	*U* = 159*^a^*	<0.001**
Visuomotor (copying)	22.72 ± 5.70	33.45 ± 3.37	*U* = 84*^a^*	<0.001**
Visual constructional (block design)	20 ± 8.61	33 ± 8.09	*t* (79) = –6.75	<0.001**
Verbal short-term memory (forward digit span)	4.52 ± 0.93	5.93 ± 1.0	*U* = 249^a^	<0.001**
Visuospatial short-term memory (block tapping)	4.42 ± 1.11	5.87 ± 1.09	*U* = 290^a^	<0.001**
Working memory (backward digit span)	3.20 ± 0.86	4.55 ± 1.21	*U* = 296 ^a^	<0.001**
Motor inhibition (Go-NoGo)	8.12 ± 4.69	5.74 ± 3.86	*t* (79) = 2.37	0.02*
Cognitive inhibition (stroop test, time index)	33.16 ± 17.31	22.58 ± 9.32	*U* = 317.5^a^	<0.001**
Planning (tower of London)	5.28 ± 1.21	6.11 ± 1.0	*U* = 470^a^	0.002*
Shifting (revised card sorting test)	2.40 ± 2.13	1.06 ± 1.29	*U* = 479^a^	0.003*
Alertness (reaction times)	416.58 ± 114.25	331.19 ± 65.20	*U* = 367.5^a^	<0.001**
Attentional vigilance (coefficient of variation)	0.32 ± 0.11	0.22 ± 0.06	*U* = 313^a^	<0.001**

Values are presented as mean ± SD (standard deviation), except for sex, laterality, and pathological ADHD-RS-IV score.

DCD, developmental coordination disorder; TD, typically developing children; F, female; M, male; Laterality, Edinburgh handedness inventory (Oldfield, 1971), R, right-handed; L, left-handed; A, ambidextrous; DCD-Q, developmental coordination disorder questionnaire (Martini et al., 2011); ADHD-RS-IV, attention-deficit/hyperactivity disorder rating scale IV (DuPaul et al., 1998); MABC-2, movement assessment battery for children, 2nd ed. (Henderson et al., 2007; Marquet-Doléac et al., 2016).

X^2^ = chi-squared test; t = two-sample t-test.

^a^Mann-Whitney U test in cases of non-normality of the data.

^b^Welch’s t-test in cases of violation of homogeneity of variances.

**p < 0.002 (0.05/21), statistical significance for motor and neuropsychological assessment using Bonferroni correction for multiple comparisons or p < 0.05 for demographic data and questionnaires; *p < 0.05, uncorrected for motor and neuropsychological assessment.

Children with DCD had to meet the four diagnostic criteria from the DSM-5 (American Psychiatric Association [APA], 2013) assessed by a multidisciplinary team, including pediatric neurologists and neuropsychologists. Motor skills were assessed using the French version of the Movement Assessment Battery for Children-2 (MABC-2; Criterion A, DSM-5; Henderson et al., 2007; Marquet-Doléac et al., 2016). Following international recommendations (Blank et al., 2019), children with an MABC-2 score below the 16th percentile were included if they met the other diagnostic criteria (exclusion: four children with an MABC-2 score ≥ 16th percentile out of the 63 children who were first enrolled as part of the DCD group; not considered as TD according to their clinical concern). Among children with DCD, 37 had severe (MABC-2 ≤ 5th percentile) and 13 moderate (6–15th percentile) motor impairments. The impact of motor impairments on the child’s life was measured using the DCD-Q and scored in the suspected or indicative range of DCD (Criterion B, DSM-5; Martini et al., 2011). A short parental anamnesis and the medical record were used to determine the early occurrence of symptoms (Criterion C, DSM-5), and the presence of any neurological condition (along with the observation of the child during the clinical assessment). The verbal comprehension index of the WISC-V (Wechsler, 2016) was used to assess intellectual functions (Criterion D, DSM-5). Co-occurring ADHD diagnosis was not an exclusion criterion (*n* = 29), as co-occurring neurodevelopmental conditions are frequent in DCD (Piek and Dyck, 2004; Dewey, 2018), and was assessed according to the DSM-5 criteria [American Psychiatric Association [APA], 2013] by a multidisciplinary team including pediatric neurologists and neuropsychologists. Methylphenidate medication for ADHD (*n* = 14) was interrupted at least 24 h before the assessments. In all participants, the severity of ADHD symptoms was measured using the ADHD-RS-IV parental questionnaire (DuPaul et al., 1998). This questionnaire comprises nine questions assessing the frequency (from “never” to “very often,” scored from 0 to 3) of each symptom of inattention based on DSM criteria, and nine questions assessing the frequency of each symptom of hyperactivity/impulsivity. The scores on the different questions are added together to obtain a total score, with value of > 28 being indicative of ADHD.

The TD children included in the study had no: history of motor difficulties, scored equal or above the 25th percentile on the global score of the MABC-2 (exclusion: four TD children out of the 40 children first enrolled as part of the TD group); (suspected) DCD based on the DCD-Q; any neurodevelopmental disability, based on the short anamnesis and ADHD-RS-IV parental questionnaire (exclusion: two TD children; DuPaul et al., 1998).

Children from both groups were excluded if they showed any intellectual disability (assessed using the verbal comprehension index of the WISC-V < 80; exclusion: four children with DCD), had ASD (exclusion: one child with DCD), were born very preterm (< 33 weeks gestational age, exclusion: two TD children), or had any history of psychiatric or neurological disorder (all assessed based a short anamnesis and the medical record, when available). The verbal comprehension index was chosen instead of the total IQ, as impairments of motor skills and executive functions can impact certain subtests (e.g., coding) and the total IQ score (Arffa, 2007; Alloway, 2010; Sumner et al., 2016). A restrictive cut-off of 80 was selected as only one subscale of the intellectual assessment was used and this cut-off corresponds to a performance below the 10th percentile, considered as poor in the rest of the manuscript.

Children with DCD were recruited through consultations with pediatric neurologists and healthcare professionals (*n* = 37), as well as from parent support groups on social media (*n* = 11) and in schools (*n* = 2). Children from the TD control group were recruited through acquaintances (*n* = 22), social media (*n* = 7), and primary schools (*n* = 2), after receiving approval of competent authorities, in the French-speaking part of Belgium. Written informed consents were obtained from all participants and their parents. The study was approved by the local Ethics Committee of the CUB Hôpital Erasme (Reference: P2018/179) and the Hôpital Universitaire des Enfants Reine Fabiola (Brussels, Belgium).

### Procedure

First, a semi-structured interview was conducted with at least one parent and the child. Information regarding the impact of motor difficulties on daily living and school productivity, any possible associated disorder or medical condition, medical and pregnancy history, and the socioeconomic status (SES) were collected. The SES was estimated with a double 6-point scale based on the addition of each 6-point scale for each parent’s education level (SES lowest score = 2, highest score = 12; adapted from Largo et al., 1989). Laterality was measured with the Edinburgh handedness inventory (Oldfield, 1971).

This was followed by a comprehensive motor and neuropsychological assessment, allowing verifying the inclusion criteria and characterizing the profiles of our participants. The assessment lasted approximately 3 h. The order between tasks was counterbalanced between participants. During the assessment, the tasks were grouped into four blocks: “intellectual functions,” “motor skills,” “praxis/visual and visuospatial perception/visuomotor and visual constructional skills,” and “executive and attentional functions” (see section 2.3 Instruments for further details). The order in which the four blocks were administered was randomized for each child. For “intellectual functions” and “motor skills,” the subtests of the WISC-V and the MABC-2 were presented in the order of the test battery. Of note, only some subtests of the WISC-V were used in further analyses to answer to some specific questions or assess the verbal comprehension index. For the last two blocks, the order was pseudo-random so as to avoid the consecutive presentation of two tests assessing related dimensions (e.g., visual and visuospatial perception, visuomotor skills, cognitive and motor inhibition). Clinical assessment was realized during one (DCD = 2/50; TD = 26/31), two (DCD = 25/50; TD = 5/31), or three (DCD = 23/50) different days to avoid a fatigue effect (mainly within 1 month; except for eight participants, due to difficulties in managing the appointments or to pandemic lock-down; 40–147 days between the first and last appointments). These sessions took place with the same investigator (DVD) in two Belgian hospitals (Brussels), the CUB Hôpital Erasme (DCD = 30/50; TD = 31/31) and the Hôpital Universitaire des Enfants Reine Fabiola (DCD = 20/50). This study was part of a longer experimental protocol, and some children (DCD = 38/50; TD = 31/31) also underwent a magnetoencephalography/electroencephalography investigation (Van Dyck et al., 2020, 2022) and a procedural learning task (Van Dyck et al., 2021).

### Instruments

#### Fine and gross motor skills

*Manual dexterity, aiming and catching, and balance (static/dynamic)* were assessed with the eight subtests of the MABC-2 (age band 2 for children aged 7–10 y.o. and age band 3 for children aged 11 y.o.; Henderson et al., 2007; Marquet-Doléac et al., 2016). Studies reported good construct and concurrent validity of the MABC-2, and good to excellent test-retest reliability (test-retest coefficients ranged from 0.79 to 0.96; Schulz et al., 2011; Griffiths et al., 2018). It has been shown that for TD children, the construct validity of the three subscales of the MABC-2 becomes stronger with the increasing age of the child. In age band 2, balance seems to be subdivided into two subfactors (static vs. dynamic). Moreover a second-order factor of general motor skills was found suggesting similar performance level across the three motor domains (i.e., manual dexterity, aiming and catching, and balance; Schulz et al., 2011).

#### Praxis

(1) *Meaningless postures.* Hand position imitation (NEPSY-2; Korkman et al., 2007): children imitate a maximum of 12 hand positions (for each hand) with an increasing complexity demonstrated by the investigator. The assessment of one hand is interrupted if the child fails three consecutive positions. Score reflects correct imitations for both hands (maximum = 24). NEPSY-2 has a good concurrent and discriminative validity (Davis and Matthews, 2010), and the manual reports good to excellent test-retest reliability for most subtests (test-retest coefficients for the sensorimotor functions ranged between 0.66 and 0.84). (2) *Meaningless gestures.* Manual motor sequences (NEPSY-2; Korkman et al., 2007): children reproduce a series of various rhythmic sequences of hand movements. Scores reflect the number of sequences correctly reproduced (maximum = 60; five trials per sequence). The task stops if the child fails all the trials of four consecutive sequences.

#### Visual and visuospatial perception

(1) *Visual perception.* Visual closure (DTVP-2; Hammill et al., 1993): children mentally supply the missing part of a figure to match a completely drawn model. Scores correspond to the number of correct answers (maximum = 20). The task stops after three errors over five trials. DTVP-2 has been reported to present good construct validity, a reasonable degree of concurrent validity, and visual closure has a good test-retest reliability (test-retest coefficient = 0.85) and item discrimination (coefficients above 0.47 for all age groups, with a coefficient > 0.3 considered as acceptable; Brown and Hockey, 2013; Hammill et al., 1993). (2) *Visuospatial perception*. Test of topological and directional relations (RTD Lacert; Lacert, 1987): children are asked to recognize bars orientation among a series of distractors. The total number of errors is measured (mirror, below, or above 18 degree), with a maximum of 22 errors. RTD Lacert is a test commonly used in clinical practice in Belgium by occupational therapists in the diagnostic assessment of children with DCD (normative data were updated in 2010; Barray, 2010; Lefevere and Alexandre, 2010).

#### Visuomotor and visual constructional

(1) *Eye-hand coordination* (DTVP-2; Hammill et al., 1993): children draw a line within a delimited band with progressive complexity (narrow bands and curves). Higher scores are attributed to drawings that remain within the band, and fewer when the trace deviates from the band (scored from 0 to 4 for each line section; maximum = 184). Eye-hand coordination has a good test-retest reliability (test-retest coefficient = 0.84) and acceptable item discrimination (coefficients above 0.31 for all age groups; Hammill et al., 1993; Brown and Hockey, 2013). (2) *Visuomotor skills*. Copying figures (DTVP-2; Hammill et al., 1993): children reproduce a maximum of 20 figures of increasing complexity. Scores reflect an addition of points attributed to figures similar (2 points) or with few alterations (1 point) compared to the model (maximum = 40). The task stops after three consecutive failed figures (0 point). Copying has a good test-retest reliability (test-retest coefficient = 0.82) and item discrimination (coefficients above 0.51 for all age groups; Hammill et al., 1993; Brown and Hockey, 2013). (3) *Visual constructional skills*. Block design test (WISC-V; Wechsler, 2016): children reproduce increasingly complex models with 3D block designs (white, red, and white/red faces). Raw scores are an addition of the points attributed to correct responses in conjunction with the time taken to complete the design for the more complex trials (maximum = 58). Efficacy research reports good construct and concurrent validity of WISC-V, and block design has a good test-retest reliability (test-retest coefficient = 0.84; Wechsler, 2014).

#### Short-term memory

(1) *Verbal short-term memory*. Forward digit span (WISC-V; Wechsler, 2016): participants recall growing sequences of digits (forward order) until they fail to recall correctly at least one of the two trials for a sequence length. Final scores correspond to the highest sequence of digit (i.e., span; maximum = 10) repeated successfully, as it enables direct comparison with the span in visuospatial short-term memory and working memory. Digit span test (WISC-V) has a good test-retest reliability (test-retest coefficient = 0.84; Wechsler, 2014). (2) *Visuospatial short-term memory*. Block tapping test (Fournier and Albaret, 2013): participants reproduce growing sequences of block tapping (forward order) until they fail to reproduce correctly at two of the three trials for a sequence length. If the first two trials are correct, the third is not administered. Final scores correspond to the highest sequence of blocks repeated successfully (maximum = 9). Corsi’s block tapping test is widely used to assess visuospatial short-term memory in clinical practice and research, and has proven its validity and reliability in clinical populations and in typical development (e.g., McLean and Hitch, 1999; Kessels et al., 2000; Farrell Pagulayan et al., 2006; de Paula et al., 2016).

#### Executive functions

(1) *Working memory*. Backward digit span (WISC-V; Wechsler, 2016): participants recall growing sequences of digits in backward order until they fail to recall correctly at least one of the two trials for a sequence length. Of note, the first two spans comprise two presentations of two trials. Final scores correspond to the highest sequence of digits repeated successfully (maximum = 8). (2) *Motor inhibition*: Go-NoGo test (Zimmermann and Fimm, 2004): children respond as quickly as possible to a target “x” (50% of trials), but not to a distractor “+.” The number of errors committed (responses to a distractor) were counted (maximum = 20). The manual reports good construct and discriminative validity, and poor to moderate test-retest reliability of different measures of the Go-NoGo test in children (correlations: 0.34–0.55). (3) *Cognitive inhibition:* Stroop test (non-reader version; Catale et al., 2014) comprises three conditions: denomination (i.e., color of squares), fruits (i.e., usual color of fruits presented in black), and interference (i.e., usual color of fruits presented in another color). The time and error for each condition are measured. The interference score, used for group comparison, is measured as the time difference between interference and denomination conditions. A lower score reflects a better performance in the interference condition. The paradigms assessing executive functions are widely used in neuropsychology and have proven their validity and reliability in many clinical populations and in typical development (e.g., Donders and Wildeboer, 2004; Su et al., 2008; Sullivan et al., 2009; Catale et al., 2014; Macdonald et al., 2014; Mary et al., 2016; Unterrainer et al., 2020). (4) *Planning.* Tower of London (child-adapted version; Shallice, 1982; Mary et al., 2016): children resolve a problem with the least number of moves as possible. The child version comprised seven trials (five neutral from three to seven moves, one negative and one positive trials with five moves). The number of trials correctly completed is recorded (maximum = 7). (5) *Shifting (mental flexibility)*. Revised Wisconsin Card Sorting Test (Nelson, 1976; Mary et al., 2016): children classify a set of 48 cards based on three dimensions (color, number, or shape). Only one dimension is used at a time, and children use the feedback from the investigator to deduce the correct dimension. After six consecutive correct matches, the dimension changes. The number of perseveration errors was used for group comparison.

#### Attentional functions

(1) *Alertness* is measured based on the median reaction times (RT) in a simple RT task (Zimmermann and Fimm, 2004). Children are asked to respond as quickly as possible to a stimulus appearing on a computer screen. The manual reports good test-retest reliability of different measures of the Alertness test in children (correlations: 0.68–0.78). (2) *Attentional vigilance:* the coefficient of variation (standard deviation/mean) of the RTs in the alertness task reflects the intra-individual variability of the participant. Standard deviations of the RTs are compared to normative data.

### Statistical analyses

#### Descriptive analyses

We first compared motor and neuropsychological scores between children with DCD and TD children using two-tailed unpaired *t*-tests to describe the global profile of the two samples at a group level. Percentiles and standard scores were used for motor assessment: percentiles for the global score of MABC-2 (as this score is part of the inclusion criteria) and standard scores (mean = 10, standard deviation = 3) for the three subscales. Raw scores were used for neuropsychological assessment. The non-parametric Mann-Whitney test was used in cases of non-normality of the data, and Welch’s *t*-test in cases of violation of homogeneity of variances. Significance level was set at *p* < 0.002, Bonferroni corrected for the number of scores (*p* < 0.05/21). Then, we performed descriptive analyses of our DCD sample by comparing the results of each child with DCD to the normative data with regard to age and sex (when available in the normative data). We measured the percentage of children with impairments in our DCD sample for each measure and in at least one subtest for each general motor, perceptual and cognitive dimension (i.e., fine and gross motor skills, praxis, visual and visuospatial perception, visuomotor and visual constructional, short-term memory, executive functions, attentional functions). Impairment was defined as a performance below the 5th percentile (set at the false-positive rate; Godefroy et al., 2014), i.e., below –1.65 standard deviation from the mean. A performance below the 10th percentile (-1.3 standard deviation) was considered as poor.

#### Clustering analyses

Before proceeding to cluster analysis, we converted our variables into standardized z-scores based on the age-matched (and sex-matched when available) normative data, as the variables were measured in different units (Milligan and Cooper, 1987; Hoare, 1994; Asonitou and Koutsouki, 2016) and to control for age effect on performance. A z-score of zero corresponded to the mean of the age-matched (and sex-matched) general population, and z-score of one to its standard deviation. The direction of the z-score was adapted to facilitate interpretation, with a positive value reflecting a higher performance. Outliers with a z-score beyond 3 standard deviations were curtailed to a value of ± 3 (e.g., Renwick et al., 2015), as clusters are sensitive to extreme values (Milligan and Cooper, 1987; Allen and Goldstein, 2013).

As variables must be selected based on the problem of interest (Bratchell, 1989), three variables assessing verbal skills that are not part of the main cognitive areas of interest of this study (i.e., verbal short-term memory, similarities, and vocabulary; Wechsler, 2016) were kept aside and used to assess the external validity of the clustering solution. Variables containing insufficient information to contribute to the clustering solution (Milligan and Cooper, 1987) or bringing redundant information (Allen and Goldstein, 2013) were identified through Pearson’s correlations between the 18 variables of interest. Three variables were excluded from clustering analysis: motor inhibition had low correlation coefficient with the rest of the variables (i.e., insufficient information; *r* < 0.30), while copying figures and attentional vigilance were highly correlated with others (i.e., redundant information; *r* > 0.70). A total of 15 variables were then selected and included in the clustering analysis (see Table 2).

**TABLE 2:** Average performance, standard deviation, and *post hoc* multiple comparisons of the five clusters for motor, perceptual, and cognitive measures.

	Cluster 1 8 DCD	Cluster 2 15DCD + 1TD	Cluster 3 11DCD	Cluster 4 15DCD + 5TD	Cluster 5 1DCD + 25TD	*Post hoc* (sig.)^a^
Manual dexterity^b^	–2.42 ± 0.16	–2.15 ± 0.53	–1.91 ± 0.54	–1.77 ± 0.84	–0.11 ± 0.86	1–5; 2–5; 3–5; 4–5; 1–4
Aiming and catching^d^	–0.92 ± 0.77	–0.62 ± 0.88	–1.33 ± 0.49	–1.12 ± 0.75	0.17 ± 0.80	1–5; 2–5; 3–5; 4–5
Balance^d^ (static and dynamic)	–1.83 ± 0.99	–1.52 ± 0.99	–1.27 ± 1.11	–0.88 ± 1.08	0.41 ± 0.59	1–5; 2–5; 3–5; 4–5
Meaningless postures^d^ (imitating hand positions)	–1.29 ± 0.63	–1.15 ± 0.88	–1.58 ± 0.7	–0.93 ± 0.81	0.18 ± 0.85	1–5; 2–5; 3–5; 4–5
Meaningless gestures^c^,^d^ (manual motor sequences)	–1.29 ± 0.5	–1.17 ± 0.82	–1.28 ± 0.59	–1.06 ± 0.78	–0.38 ± 0.63	1–5; 2–5; 3–5; 4–5
Visual perception^c^ (visual closure)	–2.2 ± 0.35	–1.31 ± 1.06	–1.61 ± 0.93	0.70 ± 0.67	1.16 ± 0.57	1–5; 2–5; 3–5; 1–4; 2–4; 3–4
Visuospatial perception^c^ (directional relations)	–1.71 ± 0.5	–1.03 ± 0.76	0.70 ± 0.61	0.24 ± 0.87	0.65 ± 0.77	1–5; 2–5; 1–3; 1–4; 2–3; 2–4
Eye-hand coordination^b^	–1.33 ± 0.59	–1.1 ± 0.89	0.15 ± 0.64	–0.7 ± 1.09	0.54 ± 0.62	1–5; 2–5; 4–5; 1–3; 2–3
Visual constructional^b^ (block design)	–1.79 ± 0.92	–0.69 ± 0.56	–0.64 ± 0.7	0.0 ± 0.71	0.63 ± 0.81	1–5; 2–5; 3–5; 1–4
Visuospatial short-term memory^d^ (block tapping)	–1.98 ± 0.87	–1.13 ± 1.21	–1.16 ± 0.90	–1.1 ± 0.78	1.06 ± 1.09	1–5; 2–5; 3–5; 4–5
Working memory (backward digit span)	–1.32 ± 0.86	–0.38 ± 1.04	–0.05 ± 0.80	–0.05 ± 1.09	0.84 ± 1.02	1–5; 2–5; 4–5; 1–4
Cognitive inhibition (stroop test, time index)	–2.67 ± 0.49	0.13 ± 0.67	–2.47 ± 0.65	–1.89 ± 1.05	–0.26 ± 0.90	1–5; 3–5; 4–5; 1–2; 2–3; 2–4
Planning^c^ (tower of London)	–2.12 ± 0.42	–1.4 ± 1.04	–1.2 ± 1.01	–0.54 ± 0.98	–0.38 ± 0.82	1–5; 2–5; 1–4
Shifting^c^ (revised card sorting test)	–1.81 ± 0.54	–0.54 ± 1.31	–0.02 ± 0.63	0.05 ± 0.98	0.28 ± 0.97	1–5; 1–3; 1–4
Alertness (reaction times)	–1.91 ± 0.54	–1.52 ± 0.81	–0.99 ± 0.54	–1.06 ± 0.60	–0.66 ± 0.72	1–5; 2–5; 1–3; 1–4

Values are presented as mean ± SD (standard deviation).

DCD, developmental coordination disorder; TD, typically developing children.

^a^Post hoc comparisons were performed to describe the characteristics of the clusters and the discriminant measures. Underlined cluster pairs: statistical significance using Bonferroni correction for multiple comparisons, p < 0.003 (0.05/15). Other cluster pairs: uncorrected p-value, p < 0.05.

^b^Welch’s one-way ANOVAs and Games-Howell post hoc analyses in cases of violation of homogeneity of variances.

^c^Non-parametric Kruskal-Wallis tests and Dwass-Steel-Critchlow-Fligner pairwise comparisons in cases of non-normality of the data.

^d^Measures that did not allow to discriminate between the clusters mainly composed of children with DCD.

As a first step, we performed three agglomerative hierarchical clustering analyses on the 81 participants (50 DCD and 31 TD together) and the 15 variables in order to determine the optimal number of clusters in the data. Agglomerative hierarchical clustering algorithms combine the more similar pairs of participants or clusters at each successive stage of the process to form a cluster hierarchy until all participants are part of the hierarchical structure (Milligan and Cooper, 1987; Gore, 2000; Jain, 2010; Rodriguez et al., 2019). We selected three well-known procedures based on squared Euclidean distance measures (Gore, 2000) and used in previous DCD clustering studies (e.g., Dewey and Kaplan, 1994; Hoare, 1994; Miyahara, 1994; Wright and Sugden, 1996; Tsai et al., 2008): (i) average linkage, (ii) complete linkage, (iii) Ward’s minimum variance. These algorithms enable to determine the number of clusters in the data by visually examining the dendrogram and coefficients (i.e., squared Euclidean distance between two clusters joined together) of several cluster solutions (Dewey and Kaplan, 1994; Gore, 2000; Yim and Ramdeen, 2015). Clustering process should stop at the first large increase of coefficient values, indicating that two dissimilar clusters have been joined together.

As a second step, we used the final Ward’s solution as an input for K-means, which is the most popular iterative partitioning clustering procedure based on Euclidean distance (Distefano and Mindrila, 2013) and has already been used in previous DCD studies (Dewey and Kaplan, 1994; Hoare, 1994; Miyahara, 1994; Tsai et al., 2008; Asonitou and Koutsouki, 2016). K-means update all clusters simultaneously, enabling the reassignment of the participants to another cluster at each iteration to propose a better fit (Gore, 2000; Jain, 2010; Rodriguez et al., 2019). By combining the two methods, we benefited from the ability of the Ward’s minimum variance to create highly homogeneous clusters by minimizing the within-cluster variance and maximizing the between-cluster variability (Milligan and Cooper, 1987; Gore, 2000; Distefano and Mindrila, 2013), and from the ability of K-means to reassign the participants and verify the stability of the clusters (Distefano and Mindrila, 2013). More details regarding clustering analyses are provided in Supplementary Material 2.

Internal validity (reliability) was checked by examining the recovery of the cluster membership obtained from the Ward’s minimum variance and K-means solutions (Milligan and Cooper, 1987). The solution is stable if a small number of children change cluster from one solution to another. The proportion of children belonging to the same cluster with each of the two methods was measured.

#### Description of the clusters

The performance of each cluster obtained with K-means was first described based on their z-scores and percentage of children with impairment. Performance was considered “impaired” with a z-score below -1.65 (i.e., corresponding to the 5th percentile) and “poor” with a z-score below –1.3 (i.e., 10th percentile) and more than 50% of the children impaired. Of note, a performance was considered “below average” with a z-score below –1 (i.e., 15th percentile), or if one of the two criteria was not met to be considered as “poor.” One-way ANOVAs and Tukey’s *post hoc* analyses were then performed with the clusters as between-subject variable and the clinical measures as dependent variable, in order to better describe the characteristics of the clusters and the discriminant measures, but should not be seen as a way to verify any hypothesis (Macnab et al., 2001; Tsai et al., 2008). Welch’s one-way ANOVAs and Games-Howell *post hoc* analyses were employed in cases of violation of homogeneity of variances, and non-parametric Kruskal-Wallis tests and Dwass-Steel-Critchlow-Fligner pairwise comparisons in cases of non-normality of the data. The significant results from *post hoc* analyses were used to characterize the differences between the clusters, with the differences being considered high when surviving to Bonferroni correction for multiple comparisons with a *p*-value < 0.003 (*p* < 0.05/15) and small with a *p*-value < 0.05.

#### External validity

External validity corresponds to the relevance of the classification obtained by the clustering algorithm (Dewey and Kaplan, 1994) and was examined by using the final cluster solution to predict other clinical characteristics (Milligan and Cooper, 1987; Gore, 2000). As before, we performed one-way ANOVAs and Tukey’s *post hoc* analyses (or equivalents in cases of violation of homogeneity of variances or normality) with the clusters as a between-subject variable and the following demographic and cognitive measures as dependent variables: age, severity of the ADHD symptoms (i.e., ADHD-RS-IV parental questionnaire; DuPaul et al., 1998), verbal short-term memory (i.e., forward digit span; Wechsler, 2016), and verbal skills (i.e., similarities and vocabulary of the WISC-V; Wechsler, 2016). A chi-squared test was performed on the sex ratio. As the measures excluded due to redundancy or reduced contribution to the cluster structure are relevant for clinical purposes, similar comparative analyses were performed on copying figures, attentional vigilance, and motor inhibition. Significance level was set at *p* < 0.008 for the cognitive measures, Bonferroni corrected for the number of scores (*p* < 0.05/6).

Statistical analyses were performed using JAMOVI (The jamovi Project, 2021) and SPSS (version 27 for Mac; IBM Corp, 2020) for the clustering analyses.

## Results

### Descriptive analyses

Demographic and clinical data are reported in Table 1. At the group level, children with DCD performed more poorly than their TD peers in all measures. Only three comparisons did not survive Bonferroni correction for multiple comparisons (i.e., motor inhibition, planning, and shifting). However, as depicted in Table 3 (last column), not all children with DCD were impaired in each measure. Among our sample of 50 children with DCD, moderate to large percentages of children were impaired in at least one measure assessing executive functions (92%), praxis (68%), attentional functions (52%), visual and visuospatial perception (46%), and visuomotor and visual constructional skills (36%). A smaller percentage was impaired in short-term memory (28%), and only 8% had poor verbal skills.

**TABLE 3:** Percentage of children with impairments for each cluster and the complete sample of children with developmental coordination disorder on the different measures compared to the normative data.

	Cluster 1 DCD (*n* = 8)	Cluster 2 DCD + TD (*n* = 16)	Cluster 3 DCD (*n* = 11)	Cluster 4 DCD + TD (*n* = 20)	Cluster 5 TD + DCD (*n* = 26)	Total DCD (*n* = 50)
**Characteristics**						
DCD/TD, *n*	8/0	15/1	11/0	15/5	1/25	50/0
Pathological ADHD-RS-IV	62.5% (5)	43.75% (7)	54.55% (6)	45% (9)	3.85% (1)	56% (28)
**Fine and gross motor skills**	**100% (8)**	**87.5% (14)**	**90.91% (10)**	**70% (14)**	**11.54% (3)**	**92% (46)**
Manual dexterity	100% (8)	87.5% (14)	81.82% (9)	65% (13)	7.69% (2)	88% (44)
Aiming and catching	12.5% (1)	12.5% (2)	36.36% (4)	30% (6)	3.85% (1)	26% (13)
Static and dynamic balance	62.5% (5)	60.5% (10)	45.45% (5)	25% (5)	0% (0)	50% (25)
**Praxis**	**62.5% (5)**	**68.75% (11)**	**63.64% (7)**	**55% (11)**	**15.38% (4)**	**68% (34)**
Meaningless postures (imitating hand positions)	50% (4)	25% (4)	45.45% (5)	15% (3)	3.85% (1)	32% (16)
Meaningless gestures (manual motor sequences)	50% (4)	56.25% (9)	54.55% (6)	45% (9)	11.54% (3)	56% (28)
**Visual and visuospatial perception**	**87.5% (7)**	**56.25% (9)**	**63.64% (7)**	**0% (0)**	**0% (0)**	**46% (23)**
Visual perceptual (visual closure)	87.5% (7)	43.75% (7)	63.64% (7)	0% (0)	0% (0)	42% (21)
Visuospatial perception (directional relations)	50% (4)	18.75% (3)	0% (0)	0% (0)	0% (0)	14% (7)
**Visuomotor and visual constructional**	**62.5% (5)**	**37.5% (6)**	**9.09% (1)**	**30% (6)**	**0% (0)**	**36% (18)**
Eye-hand coordination	37.5% (3)	37.5% (6)	0% (0)	25% (5)	0% (0)	28% (14)
Visuomotor skills (copying)	12.5% (1)	0% (0)	0% (0)	0% (0)	0% (0)	2% (1)
Visual constructional (block design)	62.5% (5)	6.25% (1)	9.09% (1)	5% (1)	0% (0)	16% (8)
**Short-term memory**	**50% (4)**	**50% (8)**	**9.09% (1)**	**5% (1)**	**0% (0)**	**28% (14)**
Verbal short-term memory (forward digit span)	12.5% (1)	31.25% (5)	9.09% (1)	0% (0)	0% (0)	14% (7)
Visuospatial short-term memory (block tapping test)	50% (4)	25% (4)	9.09% (1)	5% (1)	0% (0)	20% (10)
**Executive functions**	**100% (8)**	**100% (16)**	**90.91% (10)**	**90% (18)**	**57.69% (15)**	**92% (46)**
Working memory (backward digit span)	50% (4)	18.75% (3)	9.09% (1)	10% (2)	0% (0)	20% (10)
Motor inhibition (Go-NoGo)	50% (4)	43.75% (7)	36.36% (4)	40% (8)	34.62% (9)	42% (21)
Cognitive inhibition (Stroop test, time index)	100% (8)	0% (0)	81.82% (9)	70% (14)	11.54% (3)	52% (26)
Planning (tower of London)	100% (8)	68.75% (11)	63.64% (7)	25% (5)	19.23% (5)	58% (29)
Shifting (revised card sorting test)	50% (4)	25% (4)	0% (0)	10% (2)	3.85% (1)	18% (9)
**Attentional functions**	**100% (8)**	**75% (12)**	**9.09% (1)**	**25% (5)**	**11.54% (3)**	**52% (26)**
Alertness (reaction times)	75% (6)	75% (12)	9.09% (1)	20% (4)	11.54% (3)	46% (23)
Attentional vigilance (reaction times SD)	100% (8)	56.25% (9)	0% (0)	20% (4)	3.85% (1)	42% (21)
**Verbal skills**	**37.5% (3)**	**6.25% (1)**	**0% (0)**	**25% (3)**	**0% (0)**	**8% (4)**
Similarities	12.5% (1)	0% (0)	0% (0)	0% (0)	0% (0)	2% (1)
Vocabulary	25% (2)	6.25% (1)	0% (0)	0% (0)	0% (0)	6% (3)

Impairment was defined as a score equal or below the 5th percentile or –1.65 standard deviation (z-score) in regards of the normative data.

DCD, developmental coordination disorder; TD, typically developing; ADHD-RS-IV, attention-deficit/hyperactivity disorder rating scale IV (DuPaul et al., 1998). Bold values/characters are categories/headings.

Given the high prevalence of impaired executive functions in our sample of children with DCD compared to the literature (between 40 and 60%; Wilson et al., 2017, 2020), we compared children with and without associated ADHD to ensure that these impairments were not better explained by this associated diagnosis. Independent sample *t*-tests did not reveal any significant difference between children with DCD with (*n* = 29) and without (*n* = 21) associated ADHD on any of the executive and attentional measures (all *p*s > 0.16), thus suggesting that the high level of executive impairment in our sample was not only related to the co-occurring ADHD diagnosis.

### Clustering analyses

#### Cluster solution

The jumps in the cluster coefficients and the dendrograms of the hierarchical agglomerative clustering methods revealed that a five-cluster solution fits the data (see Supplementary Material 3). Two of the three hierarchical agglomerative clustering methods (Ward’s minimum variance and average linkage) indicated five clusters in the data, four mainly composed of children with DCD and one of TD children. The last method (complete linkage) indicated six clusters, four mainly composed of children with DCD and two of TD children. The five-cluster Ward’s solution was subjected to the K-means iterative partitioning method. A good overall recovery was observed between Ward’s and K-means methods, with a total of 97.5% of the children classified in the same cluster (two participants were reassigned: one child with DCD from cluster 2 to cluster 4 and one TD child from cluster 4 to cluster 5).

#### Description of the clusters

**FIGURE 1:**
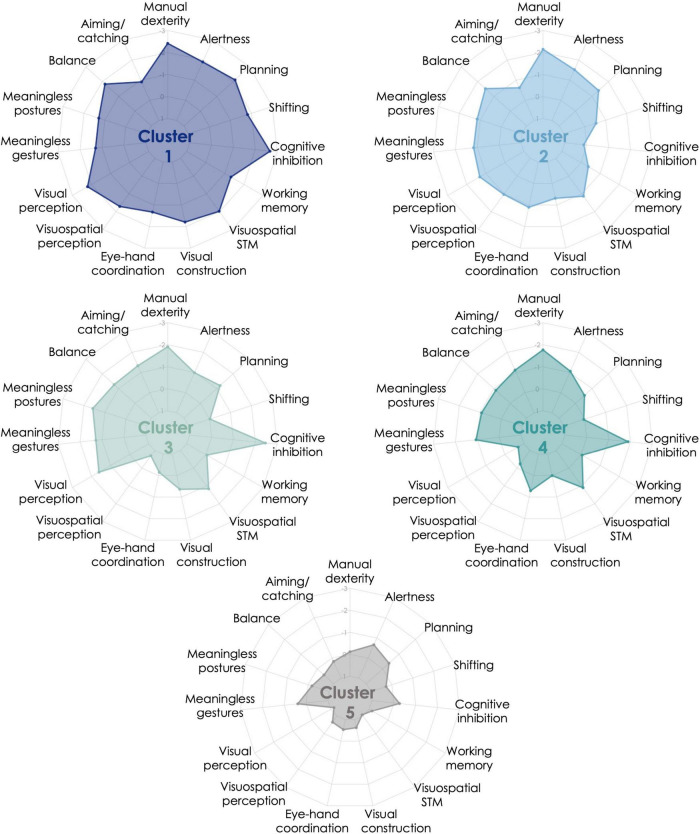
Cluster profiles from K-means iterative partitioning analysis. Performance is expressed in z-score based on the normative data available for each motor, perceptual, and cognitive measures included in the clustering analysis. Performance is expressed in z-score based on the normative data available for each cognitive and motor measures included in the clustering analysis. Five clusters resulted from K-means analysis: Cluster 1 = 8 children with developmental coordination disorder (DCD), Cluster 2 = 15 children with DCD and 1 typically developing (TD) child, Cluster 3 = 11 children with DCD, Cluster 4 = 15 children with DCD and 5 TD children, Cluster 5 = 25 TD children and 1 child with DCD.

The K-means analysis performed after Ward’s solution classified the children with and without DCD into five clusters with distinct patterns of performance on the motor, perceptual and cognitive measures. Figure 1 and Table 2 represent the profile of each cluster in each of the measures (i.e., mean performance for each measure expressed in z-score based on the normative data). Table 3 describes the percentage of children with impairments for each cluster and the complete sample of children with DCD in each motor, perceptual, and cognitive measure. The skills or functions mentioned in the cluster names correspond to those for which the cluster obtained z-score below –1.3 and for which more than 50% of the children were impaired. The main results are first described for each cluster and then compared to other clusters. One-way ANOVAs and Tukey’s *post hoc* analyses (or equivalents in cases of violation of homogeneity of variances or normality) showed that, besides subtle differences, the fine and gross motor measures and the two measures of praxis did not enable to discriminate between the clusters of children with DCD (see Table 2).

Cluster 1 (*generalized impairments*) was comprised of eight children with DCD with generalized impairments. Indeed, this cluster was characterized by impaired manual dexterity, balance, visual and visuospatial perception, visual constructional skills, visuospatial short-term memory, as well as executive (cognitive inhibition, shifting, planning) and attentional functions. The other measures were also below average, except for aiming and catching. These children performed more poorly than Cluster 5 (mainly composed of TD children) on all measures, and then Cluster 4 (mildly impaired) on most of the measures (perceptual, visual constructional, and executive and attentional functions). Cluster 1 displayed poorer shifting skills than Clusters 3, 4, and 5, and the lowest visual constructional and balance skills.

Cluster 2 (*manual dexterity, balance, planning, and attention*) was composed of 15 children with DCD and 1 TD child. This cluster was characterized by impaired manual dexterity, poor performance in balance, planning, and alertness. Children in Cluster 2 had performance below average in most motor, perceptual, and attentional measures. Except for planning, this cluster showed preserved executive functions. Compared to the other clusters, these children had lower visual perception than Cluster 4, lower visuospatial perception than Clusters 3 and 4, lower visuomotor coordination than Cluster 3, and higher cognitive inhibition than Clusters 1, 3, and 4.

Cluster 3 (*manual dexterity, inhibition, and visual perception*) was composed of 11 children with DCD. This cluster was characterized by impaired manual dexterity and cognitive inhibition, but also poor performance in visual perception. They had performance below average on most of the motor-loaded measures, while the other measures were mostly around average. The visual perception measure enabled the discrimination between this cluster and Cluster 4 and contrasted with the high visuospatial perceptual skills. According to *post hoc* analyses, this cluster also performed similarly to Cluster 5 (no impairment cluster) on executive (except for cognitive inhibition) and attentional measures, but poorer in most of the motor-loaded measures (except for eye-hand coordination). Compared to the other clusters, children in Cluster 3 had high visuospatial and visuomotor skills (higher than Clusters 1 and 2 and similar to Cluster 5). Cluster 3 exhibited the lowest performance in aiming and catching. Their low performance in imitating meaningless postures was not statistically discriminative from the three other clusters that were mainly composed of children with DCD.

Cluster 4 (*mild impairments: manual dexterity and inhibition*) included 15 children with DCD with mild impairments and 5 TD children. This cluster had impairments mainly in manual dexterity and cognitive inhibition, and performance below average in few motor-loaded measures. These children were considered as mildly impaired as most of their results were around average (above z-score –1). This cluster is close to Cluster 3, as visual perception (i.e., visual closure) is the only discriminative measure between them. Children in Cluster 4 showed better visual constructional skills, as well as visual and visuospatial perception than at least one other cluster including children with DCD and performed poorer than Clusters 2 and 5 in cognitive inhibition. They also performed similarly to Cluster 5 (*no impairment*) on planning, shifting, and attentional and perceptual measures (visual and visuospatial), but poorer in most motor-loaded measures (excepted for visual constructional skills).

Cluster 5 (*no impairment*) comprised 25 TD children and 1 child with DCD. This cluster performed around the average level on each measure and above the other clusters on most of the measures.

#### External validity

The results of the clusters on the demographic, questionnaire and six cognitive measures excluded from the cluster analysis (expressed as z-scores, calculated on normative data for each measure) and the *post hoc* comparisons between clusters are reported in Table 4. The five clusters showed significant differences in age, severity of ADHD symptoms, and some of the cognitive measures (i.e., verbal skills, verbal short-term memory, visuomotor skills, and attentional vigilance) that were not part of the clustering analysis. These differences highlight the relevance of the classification obtained with the algorithms. More precisely, Clusters 1 and 2 differed significantly from Cluster 5 on most of the measures, and from Clusters 3 and 4 on fewer measures. Clusters 3 and 4 showed similar profiles, with more severe ADHD symptoms and poorer visuomotor skills than Cluster 5. The difference in severity of ADHD symptoms mainly occurred between clusters mainly composed of children with DCD (Clusters 2, 3 and 4) and Cluster 5. However, co-occurring ADHD diagnosis (*i.e.*, assessed by a multidisciplinary team including pediatric neurologists and neuropsychologists) was equally distributed among the four clusters mainly composed of children with DCD [χ^2^(3) = 1.9; *p* = 0.59].

**TABLE 4:** Average performance, standard deviation, and *post hoc* multiple comparisons of the five clusters on demographic, questionnaire and three additional cognitive measures used to test the external validity of the clustering solution and the three measures excluded from clustering analysis due to redundancy or not contributing sufficiently to the cluster solution.

	Cluster 1 8 DCD	Cluster 2 15DCD + 1TD	Cluster 3 11DCD	Cluster 4 15DCD + 5TD	Cluster 5 1DCD + 25TD	*Post hoc* (sig.)^a^
Age	8.66 ± 0.91	8.59 ± 1.31	9.91 ± 1.56	10.5 ± 1.36	9.81 ± 1.35	1–4; 2–4; 2–5
Sex (*n* F/M)^d^	3/5	3/13	2/9	2/18	10/16	–
ADHD symptoms^b^,^d^ (ADHD-RS-IV)	29.62 ± 15.1	25.56 ± 14.0	30.54 ± 9.16	25.7 ± 13.39	12.62 ± 8.75	2–5; 3–5; 4–5
Similarities	–0.29 ± 0.77	–0.04 ± 0.88	0.33 ± 0.62	0.78 ± 1.04	0.72 ± 0.75	1–4; 2–4; 1–5; 2–5
Vocabulary	–0.67 ± 0.8	–0.35 ± 0.84	0.94 ± 0.71	0.88 ± 1.01	1.05 ± 0.82	1–3; 2–3; 1–4; 2–4; 1–5; 2–5
Verbal short-term memory (forward digit span)	–0.75 ± 0.79	–1.03 ± 0.73	–0.46 ± 0.63	–0.24 ± 0.88	0.53 ± 0.87	1–5; 2–5; 2–4; 3–5; 4–5
Visuomotor skills (copying)	–1.0 ± 0.39	–0.75 ± 0.37	–0.39 ± 0.47	–0.22 ± 0.59	0.59 ± 0.54	1–5; 2–5; 3–5; 4–5; 1–4; 2–4
Attentional vigilance^c^	–2.24 ± 0.17	–1.60 ± 0.93	–0.93 ± 0.40	–0.88 ± 0.88	–0.34 ± 0.76	1–3; 1–4; 1–5; 2–5
Motor inhibition^c^,^d^	–1.41 ± 0.73	–1.06 ± 1.38	–1.24 ± 0.86	–1.04 ± 1.15	–0.82 ± 1.03	–

Values are presented as mean ± SD (standard deviation), except for sex ratio.

DCD, developmental coordination disorder; TD, typically developing children; F, female; M, male; ADHD-RS-IV, attention-deficit/hyperactivity disorder rating scale IV (DuPaul et al., 1998).

^a^Post hoc comparisons were performed to describe the characteristics of the clusters and the discriminant measures. Underlined cluster pairs: statistical significance p < 0.05 for demographic and questionnaire measures and using Bonferroni correction for multiple comparisons for cognitive measures, p < 0.008 (0.05/6). Other cluster pairs: uncorrected p-value for cognitive measures, p < 0.05.

^b^Welch’s one-way ANOVAs and Games-Howell post hoc analyses in cases of violation of homogeneity of variances.

^c^Non-parametric Kruskal-Wallis tests and Dwass-Steel-Critchlow-Fligner pairwise comparisons in cases of non-normality of the data.

^d^Measures that did not allow to discriminate between the clusters mainly composed of children with DCD.

## Discussion

This study was aimed at characterizing the motor, perceptual, and cognitive profiles of school-aged children with DCD at the group level and in terms of subtypes. A moderate to large percentage of our sample of children with DCD had impairments in executive functions (92%), praxis (68%), attentional functions (52%), visual and visuospatial perception (46%), or visuomotor and visual constructional skills (36%). A small percentage had impairments in short-term memory (verbal or visuospatial; 28%). Clustering analysis performed on our complete sample (children with DCD and TD children) revealed that four clusters were mainly composed of children with DCD and one cluster comprised TD children and one child with DCD, thereby supporting the validity of this statistical approach to distinguish between children with developmental disorders and their TD peers. As expected, a cluster was characterized by generalized impairments (Cluster 1), a cluster by motor impairments and preserved perceptual skills (Cluster 4), and a cluster by no impairment (Cluster 5), while reduced executive functions were present across all the clusters with impairments. However, Cluster 3 had visual perceptual impairments contrasting with high visuospatial perception, and Cluster 2 was characterized by reduced performance in motor (i.e., manual dexterity and balance), planning, and attentional measures. Interestingly and contrary to our expectations, the identified clusters were mainly based on the severity of children’s perceptual and cognitive impairments, while the motor and praxis measures did not vary between clusters.

Among the clusters identified, the first one (*generalized impairments*, 8 children with DCD) was consistent with previous findings of a subtype with generalized impairments (Dewey and Kaplan, 1994; Hoare, 1994; Wright and Sugden, 1996; Macnab et al., 2001; Tsai et al., 2008; Vaivre-Douret et al., 2011; Asonitou and Koutsouki, 2016). The second cluster (*manual dexterity, balance, planning, and attention*, 15 children with DCD and 1 TD child) was characterized by impaired manual dexterity, poor performance in balance, planning, and alertness, associated with high cognitive inhibition. A subtype with poor manual dexterity, dynamic balance and planning had already been found in preschool children with DCD (Asonitou and Koutsouki, 2016). The third cluster (*manual dexterity, inhibition, and visual perception*, 11 children with DCD) was characterized by impaired manual dexterity and cognitive inhibition, but also poor performance in visual perception, contrasting with high visuospatial perception. This shows that perceptual skills are not uniformly impaired (e.g., Schoemaker et al., 2001) and that low perceptual skills are not specific to one subtype (i.e., Clusters 1 and 3 were both characterized by poor perceptual skills). The fourth cluster (*mild impairments: manual dexterity and inhibition*, 15 children with DCD and 5 TD children) was characterized by impairments in manual dexterity and cognitive inhibition. Children in Cluster 4 were considered as mildly impaired as most of their results were around average. Similarly to the children in Cluster 5, none of the children in this group failed the two purely perceptive tasks (i.e., visual and visuospatial), suggesting that preserved perceptual skills might be a good marker of this cluster. Nevertheless, they had lower performance in most motor-loaded measures compared to Cluster 5 (no impairment). This is in line with the previous observation of a subtype of children with motor production difficulties without perceptual impairment (Hoare, 1994; Macnab et al., 2001; Vaivre-Douret et al., 2011). The last cluster (*no impairment*), which mainly comprised TD children (*n* = 25; 1 child with DCD), performed better than at least one other cluster on all the measures.

As previously shown (Asonitou and Koutsouki, 2016), reduced executive functions are not specific to one cluster of children with DCD, but are rather scattered among all the clusters, with discrepancies in the nature and severity of these impairments. Discrepancies in performance between several measures are likely to be found within the DCD population at the group (Leonard et al., 2015), the subtype (Asonitou and Koutsouki, 2016) and the individual (Costini et al., 2017) levels. Our results revealed dissociations between the executive functions, with poor planning in Clusters 1 and 2 (and—to a lesser extent—3), cognitive inhibition in Clusters 1, 3, and 4, and shifting in Cluster 1 (*generalized impairments*). Such discrepancies could either reflect actual differences in terms of processes of the executive functions or be related to the task impurity. With regard to the suggestion of actual differences in terms of processes of executive functions, it has been shown that executive functions are structured into several main mental processes in adults (Diamond, 2013), but that such structuring during childhood is still subject to debate. Some authors found distinct processes as early as kindergarten (e.g., inhibition and shifting/working memory; Monette et al., 2015), while growing evidence points to an evolution from a unitary or bi-factorial model during childhood maturing toward at least three processes until adulthood (Karr et al., 2018; Laureys et al., 2022). Regarding the suggestion of task impurity as an explanation of the results, differences of performances on the various measures may be explained by non-executive factors, such as motor, verbal, visual perceptual and visuospatial skills (Miyake et al., 2000; Karr et al., 2018). Nevertheless, these differences of performances highlight the importance of considering each measure individually. The results of the child should be interpreted with caution by the clinician and cross-referenced with performance in other measures to account for the influence of these non-executive processes and with other measures assessing the same executive process to reliably assess this latter (Gärtner and Strobel, 2021). Moreover, given the relationship between executive functions on the one hand and school achievement (Best et al., 2011; Gerst et al., 2017; Oberer et al., 2018), social development (e.g., van Lier and Deater-Deckard, 2016), and behavioral and emotional regulation (e.g., Ardila, 2013; Predescu et al., 2020) on the other hand, children from Cluster 1 might experience more academic failure, social, behavioral, and emotional problems, as well as persistent difficulties despite interventions (Green et al., 2008). They might then require support from healthcare professionals over a longer period of time.

Distinct executive profiles were also highlighted in the TD population, with three subtypes showing relative weakness in specific processes (i.e., “flexibility and emotion regulation,” “inhibition,” and “working memory, organizing, and planning”; Vaidya et al., 2020). The authors found similar profiles, yet with greater impairments, in large samples of children with ADHD or ASD. This suggests that subtypes based on executive functions in several neurodevelopmental disorders are nested within the variability found in typical development and are not specific to one diagnosis. Accordingly, all three profiles were found in children with ADHD and children with ASD, but the distribution of patients was unequal across the profiles. In our results, it also appeared that the profiles highlighted by the clustering analyses were found independently of the presence or absence of a co-occurring disorder. Accordingly, co-occurring ADHD diagnosis and ADHD symptom severity were equally distributed across our four DCD clusters and did not lead to the creation of a distinct subtype. Altogether, there seems to be an overlap of cognitive profiles in the different neurodevelopmental disorders, suggesting that they might be part of a neurodevelopmental continuum characterized by various functional impairments arising from an atypical brain development (Dewey and Bernier, 2016; Morris-Rosendahl and Crocq, 2020). The nature of the co-occurring conditions in DCD is still not well understood (Visser, 2003), with some evidence that co-occurring neurodevelopmental disorders share some characteristics, such as genetic (Mosca et al., 2016; Dewey, 2018), or neural mechanisms (structural: e.g., Langevin et al., 2014; and functional: e.g., McLeod et al., 2014), but also show specific characteristics. For instance, at the cognitive level, children with ADHD have impaired working memory skills (in verbal and visuospatial domains) and preserved short-term memory, while children with DCD have greater difficulties in visuospatial short-term and working memory (Alloway, 2011). Likewise, preserved verbal skills along with low perceptual reasoning are not found in children with isolated ADHD, but are specific to children with co-occurring DCD (Parke et al., 2020). A future study including children with DCD, ADHD, and both co-occurring disorders might help to better characterize their respective cognitive profiles or on the contrary to understand continuity between the phenotypes of the diagnoses.

Regarding visual perceptual impairments, previous studies consistently found at least one subtype of children with DCD characterized by lower visual perceptual skills (Hoare, 1994; Macnab et al., 2001; Green et al., 2008; Tsai et al., 2008; Vaivre-Douret et al., 2011; Costini et al., 2017). Large inter- and intra-individual variations are, however, found among the DCD population, as not all children are impacted and differences can be found between the tasks assessed (Schoemaker et al., 2001; Van Waelvelde et al., 2004; Tsai et al., 2008). This was also reflected in our results, since we found one cluster of children with global visual and visuospatial impairments (Cluster 1) and one with specific visual perceptual impairments (Cluster 3). Children with poor visuospatial perception were exclusively classified in Clusters 1 and 2, while those with poor visual constructional skills were mainly in Cluster 1, which could correspond to the visuospatial/constructional and mixed subtypes proposed previously (Vaivre-Douret et al., 2011). However, some children classified in Clusters 1 and 2 showed preserved visuospatial or visual constructional skills, which indicates that impairments in these domains were not a prerequisite for being assigned to these clusters. These results suggest that the distinction between children with and without visual or visuospatial perceptual impairments is not as straightforward as previously proposed.

Poor fine motor skills are often associated with visual perceptual impairments (Macnab et al., 2001; Green et al., 2008; Tsai et al., 2008; Vaivre-Douret et al., 2011). However, poor fine motor skills are not specific to any DCD subtype (e.g., Hoare, 1994), as they seemed to equally affect our sample of children with DCD. Subtle, although non-significant, discrepancies in gross motor skills were found between the clusters composed mainly of children with DCD, with greater balance difficulties characterizing Clusters 1 and 2. Interestingly, balance difficulties in DCD have been associated with higher risk of obesity (Zhu et al., 2014). Although a direct measure of weight was not performed in our sample, it would be interesting to test the hypothesis of higher likelihood to suffer from obesity in children from Clusters 1 and 2. Regular physical activity should therefore be part of the clinical recommendations and intervention plan for children in the subtypes with balance problems. Previous studies also highlighted gross motor discrepancies, but worse balance does not seem to be associated with a specific performance profile (e.g., some subtypes were characterized by associated poor or, conversely high manual dexterity; Hoare, 1994; Wright and Sugden, 1996; Green et al., 2008). One possible explanation concerning the lack of distinction in motor performance between our subtypes concerns the factorial structure of motor skills in childhood (Schulz et al., 2011). It has been shown that a second-order general motor skills factor could explain a considerable part of variance in each motor domain of the MABC-2 in children aged 7–10 y.o. (i.e., over-represented in our sample). Therefore, we hypothesize that the children in our study sample have similar performance in the various motor skills assessed because of this second-order general motor skills factor.

Altogether, our results suggest that DCD is characterized by a spectrum of motor, perceptual and cognitive impairments and that children with co-occurring ADHD are not characterized by a distinct profile. The profiles of the clusters highlighted in our study tend to support the hypothesis of a dimensional approach (i.e., continuous phenotypes rather than discrete categories) of neurodevelopmental disorders (e.g., Constantino and Todd, 2003; Skuse et al., 2009; Snowling and Hulme, 2012; Balázs and Keresztény, 2014; Peters and Ansari, 2019; Drechsler et al., 2020). Accordingly, the identified clusters were mainly distinguished by quantitative rather than qualitative differences. Another promising approach proposed in the context of ASD brings together categorical and dimensional approaches (Tang et al., 2020). This so-called “mosaic” approach proposes that an individual may express one or more characteristic factors (categorical) of the disorder to varying degrees (continuous). In this view, the poor motor coordination skills of a child with DCD can be associated to certain degrees of impairment in visual perceptual skills, executive functions, and so on.

The spectrum of reduced motor, perceptual and cognitive skills in the DCD population calls for a better understanding of the underlying brain mechanisms. Disruption in specific subregions and connections belonging to the parallel cortico-striatal and cortico-cerebellar loops that underpin motor, executive, and attentional functions might be a feature of the various clusters (Clusters 1–4; Fazl and Fleisher, 2018; Guell et al., 2018; Guell and Schmahmann, 2020). Disruption of the fronto-parietal networks might underpin impairments in cognitive control of Clusters 1, 3, and 4 (Ardila et al., 2018) and stimulus-guided attention of Cluster 2 (ventral fronto-parietal network including temporo-parietal junction and ventral frontal cortex; Corbetta and Shulman, 2002). Moreover, the dorsal extrastriate visual brain system that underpins perception for action, motion detection, and visuomotor integration might be involved in Cluster 1, and the ventral extrastriate visual brain system underpinning basic visual recognition in Cluster 3 (Goodale and Milner, 1992; Milner and Goodale, 2008). Altered functional (e.g., Debrabant et al., 2013; McLeod et al., 2014; Cignetti et al., 2020) and structural (e.g., Debrabant et al., 2016; Brown-Lum et al., 2020; Grohs et al., 2021) brain connectivity and activity have already been described in children with DCD within these cortico-subcortical loops. However, assessing the relationship between functional brain architecture and specific motor, perceptual, or cognitive symptoms of DCD would help to better characterize the brain networks involved in the pathophysiology of their clinical manifestations. Such a study would require larger samples of children with DCD to allow proper correlational analyses.

Including a broader range of measurements relating to perception and motor skills might improve the characterization of subtypes among those children who mainly display low motor execution and relatively well-preserved perceptual skills (Clusters 3 and 4). Indeed, previous studies also included measures such as running, kinesthesis, or transitive gestures and found dissociations between perceptive skills (kinesthesis vs. visual perception), gross motor skills (running vs. balance; Hoare, 1994; Macnab et al., 2001), and between motor execution and planning (coordination and transitive gestures vs. motor sequences; Dewey and Kaplan, 1994). Similarly, static and dynamic balance could be considered as two distinct motor skills as factor analyses have separated them in TD children aged 7–10 y.o. (i.e., the age group predominantly represented in elementary school; Schulz et al., 2011) and dissociations between these skills have already been observed between clusters in previous studies (e.g., Tsai et al., 2008). The motor functions included in this study are quite limited, as they do not allow assumptions to be made regarding the nature of the underlying mechanism and do not differentiate between clusters of children with DCD. The two praxis elements included (i.e., meaningless postures and gestures) only highlighted subtle differences between the clusters of children with DCD, although these clusters performed poorer than the cluster with no impairment. These measures might not be pure enough to discriminate between subtypes, as they involve motor production, visual analysis, and knowledge of the body schema (Costini et al., 2017). Other tasks might thus be necessary to distinguish between DCD populations, such as more complex sequential gestures (Dewey and Kaplan, 1994), gesture-related conceptual knowledge (Costini et al., 2017), or tasks assessing cerebellar function (e.g., finger-to-nose or finger tapping tests; Tran et al., 2020).

Finally, it appeared that children from Clusters 1 and 2 were younger than those from Cluster 4 with mild impairments. This age difference might reflect an evolution in the profiles of children with DCD. A longitudinal study would enable to assess the stability of these clusters and better characterize the trajectory of the profiles of children with DCD. While reduced motor skills and executive functions tend to persist in most children with DCD (no longitudinal data were found concerning the evolution of perceptual skills in children with DCD), it might be hypothesized that the severity of each symptom evolve with time (Bernardi et al., 2018; Wilson et al., 2020; McQuillan et al., 2021). For example, in ASD, symptom severity follows distinct trajectories, with a continuous improvement in 27% of the children, and a period of improvement followed by a plateau in the remaining 73% (Georgiades et al., 2021). Children with DCD might follow different trajectories, and some of them might move from one cluster to another. The impact of the interventions on the severity of the difficulties should also be considered. Another hypothesis is that certain profiles (e.g., more complex, or severe) are detected earlier. Clustering analyses on a sample composed exclusively of newly diagnosed children will help to better explain the age difference between subtypes and to distinguish between these two hypotheses. A final hypothesis is that, as a single task may measure different subprocesses of a function or different functions intermixed (Diamond, 2020) and some functions (e.g., executive functions) are still maturing during childhood (Brocki and Bohlin, 2004), children of different ages may use different strategies to respond to the task. These different strategies or subprocesses may be impaired differently according to the age of the child, and consequently according to the cluster membership.

### Implications

There are several implications that emanate from our study for clinical practice and future research. First, the discrepancies in perceptual and cognitive impairments between clusters stress the importance of carrying out a systematic and exhaustive assessment including non-motor measures when assessing a child with suspected DCD. Assessing the child’s overall functioning enables all their strengths and weaknesses to be taken into consideration during subsequent interventions. Secondly, adopting a dimensional approach of DCD in research while investigating multiple correlates associated with multiple clinical presentations will enhance our understanding of the disorder (Peters and Ansari, 2019; Tang et al., 2020). For instance, assessing systematically the main motor skills, visual perceptual skills, and cognitive functions impaired in DCD would enable to perform correlational analyses with brain functional and structural features, genetic and cognitive components. Such correlations would help to determine whether a phenotype is more likely explained by some specific brain, genetic, or cognitive factors hypothesized to be involved in the etiology of the disorder rather than others. Characterizing the intra-group variability of the samples recruited by describing the degrees of impairment in the perceptual skills and cognitive functions frequently associated with DCD could help explain discrepancies between studies and eventually determine for which children intervention programs are the most effective.

### Limits

Our analyses reproduced some findings from previous studies that aimed to characterize the clinical heterogeneity of DCD using statistical approaches (e.g., a cluster with generalized impairments, clusters with poor visual or visuospatial perception, and poor executive functions scattered among the clusters; Dewey and Kaplan, 1994; Hoare, 1994; Macnab et al., 2001; Tsai et al., 2008; Vaivre-Douret et al., 2011; Asonitou and Koutsouki, 2016). However, the distinct measures and underlying dimensions assessed failed to directly reproduce and validate the previously established structure of subtypes. An initial limitation of this study therefore relates to the nature of clustering analysis, which is an exploratory approach that needs replication on another sample in order to be generalized to the DCD population (Milligan and Cooper, 1987; Gore, 2000). Recruiting a larger sample size might thus be required to validate these results and enable comparisons between clusters to be made with high statistical power. This classification method can only describe exclusive clusters, and not clusters with overlapping characteristics. A further limitation of the study is that some measures from the clinical assessment failed to discriminate between clusters, such as motor inhibition (i.e., commission errors in the Go-NoGo task; Zimmermann and Fimm, 2004). The equiprobable ratio of the Go and NoGo trials might not elicit sufficient prepotent activity and consequent significant inhibitory responses (Wessel, 2018) to properly distinguish between clusters. Moreover, neuropsychological measures suffer from task impurity, as they are sensitive to multiple factors, including visual perceptual and motor skills, which might impact the specificity of our results and cluster classification. Measures enabling to dissociate specific processes might be needed to help better characterize the profiles of DCD. Finally, some of the 31 TD children in our study also had reduced executive performance in several measures (see Table 3). Interindividual variability on executive functions is expected in typical development (e.g., Lensing and Elsnere, 2018). These reduced executive functions might explain the misclassification of six TD children in clusters mainly composed of children with DCD (i.e., low planning for one TD child classified in Cluster 2 and low cognitive inhibition for the five TD children classified in Cluster 4). Misclassification is, however, a common phenomenon for mildly impaired children with DCD and their TD peers (e.g., Dewey and Kaplan, 1994; Asonitou and Koutsouki, 2016).

## Conclusion

This study was aimed at characterizing the clinical heterogeneity of DCD and revealed five main clusters among a sample of DCD and TD children based on motor, perceptual, and cognitive measures. These clusters were characterized by (i) generalized impairments, (ii) impaired manual dexterity, poor balance, planning, and attentional functions, (iii) impaired manual dexterity, cognitive inhibition, and poor visual perceptual skills, (iv) impaired manual dexterity and cognitive inhibition, and (v) no impairment. Reduced executive functions were present across the clusters of children with DCD, but the nature and severity of these impairments differed between subtypes. These results highlight the importance of assessing exhaustively the perceptual and cognitive skills of children with suspected DCD. At a more general level, this study supports the hypothesis of a dimensional approach (i.e., continuous phenotypes rather than discrete categories) of neurodevelopmental disorders (e.g., Balázs and Keresztény, 2014; Drechsler et al., 2020).

## Data availability statement

The raw data supporting the conclusions of this article will be made available by the authors, upon request to the corresponding author and after acceptance by the institutional authorities (CUB Hôpital Erasme and Université libre de Bruxelles).

## Ethics statement

The studies involving human participants were reviewed and approved by the Ethics Committee of the CUB Hôpital Erasme (Reference: P2018/179) and the Hôpital Universitaire des Enfants Reine Fabiola (Brussels, Belgium). Written informed consent to participate in this study was provided by the participants’ legal guardian/next of kin.

## Author contributions

DV designed the study, performed the investigation, analyzed the data, and wrote and reviewed the manuscript. SB and AA designed the study, wrote and reviewed the manuscript, and obtained funding. XD designed the study, supervised the project, wrote and reviewed the manuscript, and obtained funding. ND designed the study, analyzed the data, supervised the project, wrote and reviewed the manuscript, and obtained funding. All authors contributed to the article and approved the submitted version.
