# Supplementary material for: Cognitive, perceptual, and motor profiles of school-aged children with developmental coordination disorder
Source: Front Psychol. 2022 Aug 3;13:860766. doi: 10.3389/fpsyg.2022.860766 (PMC9381813; doi:10.3389/fpsyg.2022.860766)
Supplement: Supplementary file 1 [file Data_Sheet_1.docx]

Supplementary Material

# Supplementary table: Subtype classification of developmental coordination disorder in previous studies

| **Study** | **Participants** | | **Measures** | | **Comparison** | | **Algorithms** | | **Subtypes** | |
| --- | --- | --- | --- | --- | --- | --- | --- | --- | --- | --- |
| **Hoare, 1994     Macnab et al., 2001 (replication)** | 80 DCD  (+20 TD) 6-9 y.o.    62 DCD 7-12 y.o. | | • Kinesthetic acuity,  • Visual perception,  • Visuomotor integration (Beery VMI), • Manual dexterity (H: Purdue pegboard, M: BOTMP), • Static balance (H: MAND, M: TOMI), • Running (H: 50-yard dash, M: BOTMP). *H = Hoare, M = Macnab.* | | DCD mean performance | | Hierarchical (Ward’s minimum variance^a^, centroid, average linkage, complete linkage) + iterative (K-means) | | 1. Below average running and kinesthetic acuity, along with above average manual dexterity and static balance (H: 27%, M: 13%), 2. Above average visual perceptual/visuomotor skills (H: 25%, M: 17%),  3. Generalized perceptuo-motor impairments (i.e., below average visual perception, visuomotor skills, manual dexterity, kinesthetic acuity, static balance; H: 19%, M: 23%), 4. Below average visual perceptual skills, with above average manual dexterity, kinesthetic acuity, and running (H: 17%, M: 32%), 5. Motor execution problems (i.e., below average manual dexterity, static balance and running, with above average kinesthetic acuity; H: 10%, M: 15%). | |
| **Dewey & Kaplan, 1994** | 51 at risk of DCD (referenced by teachers, -1SD on screening test) + 51 TD 6-11 y.o. | | • Transitive gestures, • Motor sequencing,  • Balance (BOTMP),  • Bilateral coordination (BOTMP), • Upper limb coordination (BOTMP). | | Predicted result based on age | | Hierarchical (Ward’s minimum variance, average linkage, complete linkage) + iterative (K-means) | | 1. Generalized impairments (21% of DCD + 2% of TD),  2. Reduced balance, coordination (upper limbs and bilateral), and gestural performance (41% of DCD),  3. Reduced motor sequencing (23% of DCD), 4. No impairment (16% of DCD + 98% of TD). | |
| **Miyahara, 1994** | 55 children with learning disabilities (writing, reading, calculating) 8-14 y.o. | | Gross motor (BOTMP):  • Running speed and agility, • Balance,  • Bilateral coordination,  • Strength,  • Upper limb coordination (ball skills) | | Age-related normative data | | Hierarchical (Ward’s minimum variance, average linkage) + iterative (K-means) | | 1. No motor impairment: above average balance and balls skills, running and bilateral coordination slightly below average (44%), 2. Below average coordination (poor running speed, balance, bilateral coordination, ball skills), with around average coordination-free strength (25%), 3. Above average balance, with below average motor skills on the other measures (24%), 4. Below average balance, along with above average strength and ball skills (7%). | |
| **Study** | | **Participants** | | **Measures** | | **Comparison** | | **Algorithms** | | **Subtypes** |
| **Wright & Sugden, 1996** | | 69 at risk of DCD (MABC checklist <pc5 or <pc15) 6-9 y.o. | | MABC + MABC Checklist --> factor analysis, 5 factors: • Changing environment, • Dexterity (fast hands), • Catching,  • Dynamic balance, • Control of self. | | Transformed data from factor analysis | | Ward’s minimum variance, average linkage, complete linkage | | 1. Mild impairments: above average adaptation to environment and catching, other scores around average (61%), 2. Below average catching, with above average control of self in stable environment (14%), 3. Generalized impairments: all scores around or below average of children with motor problems or at risk (17%), 4. Below average skills to move their hands quickly, dynamic balance, and adapt to changing environment, with above average catching (7%). |
| **Tsai et al., 2008** | | 178 at risk of DCD (MABC < pc 5) 9-10 y.o. | | • MABC-2 (8 subtests), • Visual perceptual skills (TVPS-R) | | DCD mean performance | | Hierarchical (Ward’s minimum variance, centroid, average linkage) + iterative (K-means) | | 1. Below average manual dexterity, static balance, and visual perceptual skills, with above average ball skills (35%), 2. Below average manual dexterity (except for one subtest), ball skills, and static balance, with above average visual perceptual skill and dynamic balance (28%), 3. Below average ball skills, with above average visual perceptual skills, manual dexterity (except for one subtest), and static and dynamic balance (33%), 4. Generalized impairments (except for ball skills; 4%). |
| **Green et al., 2008** | | 90 DCD  5-14 y.o. | | • Manual dexterity (MABC-2), • Static balance (MABC-2), • Dynamic balance (MABC-2), • Kinesthetic acuity (COMPS),  • Visual perception (Beery VMI), • Visuomotor integration (Beery VMI). | | DCD mean performance | | Hierarchical (Ward’s minimum variance) + iterative (K-means) | | 1. Above average perceptuo-motor skills: performance around or above average on all the measures (38%), 2. Below average static balance, above average perceptual skills, fine-motor skills and dynamic balance (14%), 3. Below average static and dynamic balance, with weakness in visual perception and visuomotor skills (11%), 4. Below average perceptual skills and fine motor skills (visual perception, visuomotor skills, kinesthetic acuity, and manual dexterity), with above average balance (24%), 5. Generalized impairments: below average performance in each measure (12%). |

| **Study** | **Participants** | **Measures** | **Comparison** | **Algorithms** | **Subtypes** |
| --- | --- | --- | --- | --- | --- |
| **Vaivre-Douret et al., 2011** | 43 DCD 5-15 y.o. | *49 measures, main areas:  •* Visual/visuospatial perception,  • Visuomotor integration, • Executive functions, • Handwriting, • Language, • Kinesthetic perception, • Neuropsychomotor (NP-MOT), • Neurovisual. | Age-related normative data | Clinical characterization + Hierarchical (Ward’s minimum variance) | 1. “Ideomotor”: impaired motor programming and planning (imitation of gestures and digital praxis) and in postural control (balance, hypotonia), with preserved visual perceptual skills (12%), 2. “Visuospatial/constructional”: impaired visuospatial structuring, visuomotor integration, and visual constructional (block designs) skills, and below average visuospatial attention (39%), 3. “Mixed”: generalized impairments, mix of the two other subtypes (49%). |
| **Pieters et al., 2015** | 102 DCD + 73 mathematical learning disabilities (MLD) + 99 DCD+MLD + 136 TD | *Mathematics*:  • Semantic memory (TTR)  • Procedural skills (KRT-R, TEDI-MATH)  *Motor and visuomotor integration*:  • MABC-2  • Beery VMI | Whole-sample mean performance by school grade | Model-based clustering analysis. 3 different clustering analyses:  • Motor skills,  • Motor and visuomotor,  • Mathematics | *Motor*: 1 cluster  *Motor and visuomotor*: 3 clusters  Average or below average motor skills on both M-ABC-2 and VMI (53 % of the complete sample),  Above average motor and visual-motor integration performers (28 %),  Severe motor and visual motor integration problems (19 %).  *Mathematics*: 3 clusters   1. Average scores for both domains and had no mathematical problems (53 %), 2. Below average for both number fact retrieval and procedural calculation (29 %), 3. Below average scores for procedural calculation but not for number fact retrieval (18 %). |

| **Study** | **Participants** | **Measures** | **Comparison** | **Algorithms** | **Subtypes** |
| --- | --- | --- | --- | --- | --- |
| **Asonitou et al., 2016** | 54 DCD + 54 TD 5-6 y.o. | *Motor measures (MABC-2):* • Static balance (2 measures), • Dynamic balance (2), • Manual dexterity (4), *Cognitive measures:* • Attention (3),  • Simultaneous coding (2), • Planning (2). | Age-related normative data | Hierarchical (Ward’s minimum variance, centroid, complete linkage) + iterative (K-means) | 1. At risk: reduced jumping, and minor difficulties with manual dexterity and simultaneous coding (7% of DCD + 9% of TD), 2. Reduced manual dexterity, planning, and simultaneous coding (46% of DCD + 2% of TD), 3. Reduced manual dexterity, static/dynamic balance, and planning (22% of DCD), 4. Generalized impairment: severe reduced performance in most measures (9%), 5. No impairment: performance around average in most of the measures (manual dexterity, balance, planning), attention and simultaneous coding slightly below average (15% of DCD + 65% of TD), 6. No impairment: performance around or above average in most of the measures (24% of TD). |
| **Costini et al., 2017^b^** | 27 DCD + 100 TD (normative data) 7-13 y.o. | • Conceptual knowledge  (4 measures),  • Representational gestures (3), • Meaningless postures (3), • Meaningless sequences (2),  • Real tool use (2), • Visual constructional (2), • Visual/visuospatial perception (2), • Executive functions (3), • Intellectual efficiency and comprehension (3). | Individual score compared to age-related normative data | Multiple case study | 1. Visual: impaired visual and visuospatial perceptual skills, visual constructional skills, representational gestures, meaningless postures and sequences, and verbal skills (30%), 2. Executive functions: impaired executive functions (cognitive inhibition), representational gestures, meaningless postures and sequences (22%), 3. Gesture knowledge: impaired conceptual knowledge, representational gestures, meaningless postures and sequences, verbal comprehension (15%),  4. Others: heterogeneous subtype without impairment in visual/visuospatial perceptual skills, executive functions or conceptual knowledge (33%). |

| **Study** | **Participants** | **Measures** | **Comparison** | **Algorithms** | **Subtypes** |
| --- | --- | --- | --- | --- | --- |
| **Lust et al., 2022** | 98 DCD  8:1 ± 2:3 y.o. (mean ± SD; years:months) | • Manual dexterity (MABC-2), • Static balance (MABC-2), • Dynamic balance (MABC-2), • Visuomotor integration (Beery VMI), • Visual perception (Beery VP),  • Motor coordination (Beery MC), • Verbal IQ (WISC-III),  • Performance IQ (WISC-III). | DCD mean performance | Algorithm based on Euclidean distance (unspecified) | 1. Around or below average performance in all measures (38%), 2. Around average ball skills and balance (in at risk range relative to normative data); above average visuomotor skills, visual perception and motor coordination (25%), 3. Generalized impairment: performance around or below average in all measures and more severe than cluster 1 (18%), 4. Below average fine motor skills (manual dexterity and motor coordination), visual perception, performance IQ; above average balls skills and balance (19%). |
| *Abbreviations*: DCD = developmental coordination disorder, TD = typically developing, MLD = mathematical learning disability, MABC-2 = Movement assessment battery for children, 2nd edition, (Henderson et al., 2007); Beery VMI = Beery developmental test of visual-motor integration (Beery, 1967), Beery VP = Beery visual perception (additional test), Beery MC = Beery motor coordination (additional test); BOTMP = Bruininks-Oseretsky test of motor proficiency (Bruininks, 1978); MAND = McCarron assessment of neuromuscular development (McCarron, 1982); TOMI = Test of motor impairment (Stott et al., 1984); COMPS = Clinical observations of motor and postural skills (Wilson et al., 1994), NP-MOT = battery for neuro-developmental psychomotor functions (Vaivre-Douret, 2006); SD = standard deviation. ^a^ Underline algorithm: selected method by the authors. Two underline algorithms: solution from hierarchical agglomerative method subjected to the K-means iterative partitioning method. ^b^ Subtypes characterized based on multiple case analyses and not clustering analysis. | | | | | |

# Detailed methods: clustering analyses

Clustering analysis is an unsupervised classification method that identifies groups of objects (in this case, participants) as being more similar to each other than objects in different groups or clusters (Bratchell, 1989 ; Gore, 2000; Rodriguez et al., 2019). Several algorithms exist and can lead to different clusters, so it is recommended to replicate the clustering solution to control the internal validity (reliability) and robustness of the clusters (Gore, 2000). A combination of two main approaches of clustering analyses described in the DCD literature were used in this study on the 81 participants (50 DCD and 31 TD together) and the 15 selected and standardized variables (cognitive, perceptual and motor measures) to benefit from the advantages of the two clustering methods (Distefano & Mindrila, 2013).

As a first step, we performed three agglomerative hierarchical clustering analyses to determine the optimal number of clusters in the data. Agglomerative hierarchical clustering analysis enables a determination of the number of clusters in the data based on a visual inspection of the dendrogram and an examination of the coefficients of several cluster solutions (Dewey & Kaplan, 1994 ; Gore, 2000). Agglomerative hierarchical clustering algorithms combine the more similar pairs of clusters and participants at each successive stage of the process to form a cluster hierarchy and continues to do so until all participants are part of the hierarchical structure (Gore, 2000 ; Jain, 2010 ; Milligan & Cooper, 1987 ; Rodriguez et al., 2019). Participants are first considered as individual clusters and then sequentially linked together according to their similarity. When two participants are linked together as part of a same cluster, they won’t separate in any subsequent stage. Many linkage criteria can be found in the literature. We selected three well-known procedures based on squared Euclidean distance measures (i.e., measure the root-mean-square difference across all 15 variables between two participants; Gore, 2000) and previously used in DCD clustering studies (e.g., Dewey & Kaplan, 1994; Hoare, 1994; Miyahara, 1994; Tsai et al., 2008; Wright & Sugden, 1996): (i) average linkage (i.e., joining participants to the previously defined cluster based on the average degree of similarity between this participant and the participants included in the cluster), (ii) complete linkage (i.e., the participant with the smallest distance with the most remote participant of the cluster joins the cluster), (iii) Ward’s minimum variance (i.e., the participant joins the cluster based on the squared sum of the distances with the cluster mean and results in the smallest increase in the sum of squares in the cluster; for a detailed description of these methods, see Gore, 2000). The number of clusters is determined by visually examining the dendrogram and the coefficients. A cut-off is placed on the dendrogram to eliminate vertical lines with large values (i.e., long horizontal lines, reflecting the large distance between two clusters; Yim & Ramdeen, 2015). Coefficients are values reflecting the squared Euclidean distance between two clusters joined together. Small coefficient values indicate that the two clusters joined together are quite homogeneous. The first large increase of coefficient values indicates that two dissimilar clusters have been joined together and that it would be ideal to stop the clustering process (Gore, 2000; Yim & Ramdeen, 2015). After determining the optimal number of cluster, Ward’s method was used in the subsequent steps as it creates highly homogeneous clusters (Distefano & Mindrila, 2013) and is more accurate than the other hierarchical algorithms (Gore, 2000).

As a second step, we used the final Ward’s solution as an input for the iterative partitioning clustering (K-means) procedure (Distefano & Mindrila, 2013). Iterative partitioning clustering analysis presents an advantage of proceeding by iteration and updating all clusters simultaneously (Gore, 2000 ; Jain, 2010 ; Rodriguez et al., 2019). Participants can be reassigned to another cluster at each of the subsequent stages of the process to propose a better fit. The distance between the participants and the center of the cluster (i.e., centroid) is measured and re-evaluated at each step (i.e., each iteration). K-means is the most popular partitioning method based on Euclidean distance, and was already used in previous DCD studies (Asonitou & Koutsouki, 2016 ; Dewey & Kaplan, 1994 ; Hoare, 1994 ; Miyahara, 1994 ; Tsai et al., 2008). As the number of clusters is predetermined by the researcher in partitional algorithms, we performed the K-means algorithm after determining the number of clusters with the agglomerative hierarchical algorithms. We used Ward’s final solution as an initial partition of the data set to avoid the K-means algorithm to start with random cuts in the data because it might have produced a suboptimal solution (Distefano & Mindrila, 2013). The combination of the two methods gave the benefit of the ability from Ward’s minimum variance to minimize the within-cluster variance and maximize the between-cluster variability (Gore, 2000; Milligan & Cooper, 1987), and from the ability of K-means to reassign the participants and verify the stability of the clusters (Distefano & Mindrila, 2013). One-way ANOVAs and Tukey’s post-hocs were performed with the clusters as between-subject variable and the clinical measures as dependent variable, to better describe the characteristics of the clusters and the discriminant measures, but should not be seen as a way to verify any hypothesis (Macnab et al., 2001; Tsai et al., 2008).

# Dendrogram and cluster coefficient

## Ward’s minimum algorithm

| Agglomeration Schedule | | | | | | | |
| --- | --- | --- | --- | --- | --- | --- | --- |
| Stage | Cluster Combined | | Coefficients | Stage Cluster First Appears | | Next Stage |  |
|  | Cluster 1 | Cluster 2 |  | Cluster 1 | Cluster 2 |  |  |
| 1 | 52 | 56 | 2,042 | 0 | 0 | 5 |  |
| 2 | 66 | 80 | 4,750 | 0 | 0 | 28 |  |
| 3 | 74 | 76 | 7,751 | 0 | 0 | 37 |  |
| 4 | 32 | 38 | 10,761 | 0 | 0 | 25 |  |
| 5 | 52 | 65 | 14,233 | 1 | 0 | 29 |  |
| 6 | 5 | 33 | 17,733 | 0 | 0 | 42 |  |
| 7 | 19 | 44 | 21,396 | 0 | 0 | 36 |  |
| 8 | 23 | 40 | 25,065 | 0 | 0 | 31 |  |
| 9 | 22 | 48 | 28,756 | 0 | 0 | 23 |  |
| 10 | 59 | 62 | 32,480 | 0 | 0 | 35 |  |
| 11 | 9 | 21 | 36,340 | 0 | 0 | 18 |  |
| 12 | 2 | 43 | 40,457 | 0 | 0 | 32 |  |
| 13 | 61 | 77 | 44,660 | 0 | 0 | 35 |  |
| 14 | 3 | 18 | 48,961 | 0 | 0 | 39 |  |
| 15 | 63 | 81 | 53,287 | 0 | 0 | 19 |  |
| 16 | 53 | 71 | 57,618 | 0 | 0 | 24 |  |
| 17 | 54 | 67 | 62,551 | 0 | 0 | 33 |  |
| 18 | 9 | 15 | 67,517 | 11 | 0 | 40 |  |
| 19 | 55 | 63 | 72,825 | 0 | 15 | 29 |  |
| 20 | 70 | 79 | 78,264 | 0 | 0 | 51 |  |
| 21 | 6 | 12 | 83,996 | 0 | 0 | 36 |  |
| 22 | 37 | 78 | 89,940 | 0 | 0 | 46 |  |
| 23 | 4 | 22 | 95,925 | 0 | 9 | 39 |  |
| ... |  |  |  |  |  |  |  |
| 64 | 51 | 52 | 500,620 | 43 | 59 | 72 |  |
| 65 | 5 | 24 | 518,751 | 56 | 50 | 70 |  |
| 66 | 11 | 37 | 537,050 | 0 | 46 | 71 |  |
| 67 | 16 | 20 | 555,431 | 57 | 58 | 71 |  |
| 68 | 60 | 69 | 575,811 | 28 | 51 | 76 |  |
| 69 | 13 | 58 | 596,256 | 53 | 52 | 74 |  |
| 70 | 5 | 8 | 616,904 | 65 | 55 | 73 |  |
| 71 | 11 | 16 | 642,701 | 66 | 67 | 75 |  |
| 72 | 51 | 59 | 669,910 | 64 | 35 | 74 |  |
| 73 | 5 | 27 | 700,298 | 70 | 54 | 76 |  |
| 74 | 13 | 51 | 730,705 | 69 | 72 | 80 |  |
| 75 | 7 | 11 | 768,114 | 62 | 71 | 78 |  |
| 76 | 5 | 60 | 813,058 | 73 | 68 | 77 |  |
| 77 | 5 | 6 | 874,623 | 76 | 63 | 79 |  |
| 78 | 1 | 7 | 950,493 | 60 | 75 | 79 |  |
| 79 | 1 | 5 | 1110,113 | 78 | 77 | 80 |  |
| 80 | 1 | 13 | 1586,368 | 79 | 74 | 0 |  |


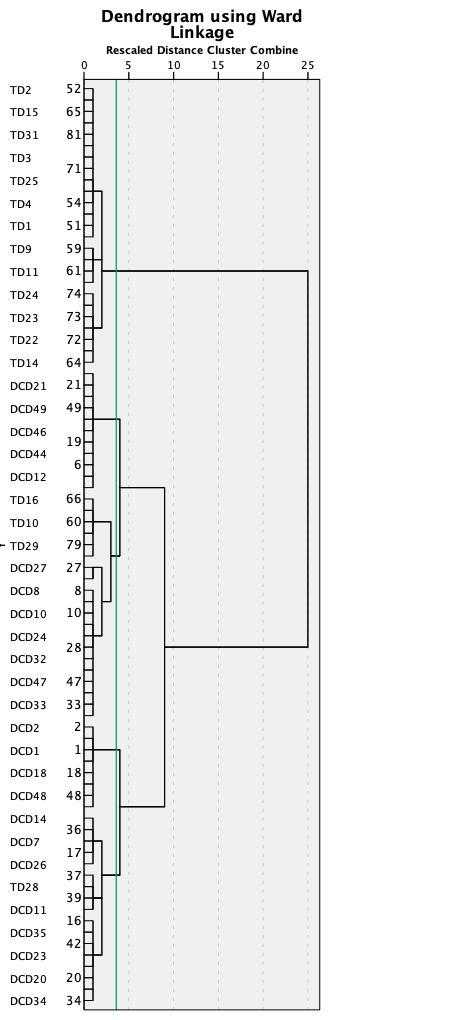


## Complete linkage algorithm

| Agglomeration Schedule | | | | | | |  |
| --- | --- | --- | --- | --- | --- | --- | --- |
| Stage | Cluster Combined | | Coefficients | Stage Cluster First Appears | | Next Stage | |
|  | Cluster 1 | Cluster 2 |  | Cluster 1 | Cluster 2 |  |  |
| 1 | 52 | 56 | 4,084 | 0 | 0 | 5 | |
| 2 | 66 | 80 | 5,416 | 0 | 0 | 42 | |
| 3 | 74 | 76 | 6,002 | 0 | 0 | 32 | |
| 4 | 32 | 38 | 6,019 | 0 | 0 | 20 | |
| 5 | 52 | 65 | 6,932 | 1 | 0 | 19 | |
| 6 | 5 | 33 | 7,000 | 0 | 0 | 44 | |
| 7 | 19 | 44 | 7,326 | 0 | 0 | 36 | |
| 8 | 23 | 40 | 7,337 | 0 | 0 | 25 | |
| 9 | 22 | 48 | 7,382 | 0 | 0 | 26 | |
| 10 | 59 | 62 | 7,448 | 0 | 0 | 34 | |
| 11 | 9 | 21 | 7,720 | 0 | 0 | 18 | |
| 12 | 2 | 43 | 8,235 | 0 | 0 | 29 | |
| 13 | 61 | 77 | 8,407 | 0 | 0 | 34 | |
| 14 | 3 | 18 | 8,602 | 0 | 0 | 26 | |
| 15 | 63 | 81 | 8,651 | 0 | 0 | 22 | |
| 16 | 53 | 71 | 8,662 | 0 | 0 | 30 | |
| 17 | 54 | 67 | 9,866 | 0 | 0 | 33 | |
| 18 | 9 | 15 | 9,958 | 11 | 0 | 37 | |
| 19 | 52 | 75 | 10,329 | 5 | 0 | 30 | |
| 20 | 32 | 47 | 10,763 | 4 | 0 | 36 | |
| 21 | 70 | 79 | 10,878 | 0 | 0 | 42 | |
| 22 | 55 | 63 | 11,185 | 0 | 15 | 48 | |
| 23 | 6 | 12 | 11,464 | 0 | 0 | 46 | |
| ... |  |  |  |  |  |  | |
| 64 | 20 | 37 | 29,591 | 54 | 45 | 70 | |
| 65 | 51 | 52 | 29,847 | 43 | 62 | 72 | |
| 66 | 58 | 59 | 31,925 | 47 | 34 | 72 | |
| 67 | 5 | 24 | 32,332 | 51 | 50 | 68 | |
| 68 | 5 | 27 | 35,066 | 67 | 56 | 75 | |
| 69 | 6 | 36 | 35,380 | 63 | 31 | 75 | |
| 70 | 11 | 20 | 38,885 | 0 | 64 | 74 | |
| 71 | 13 | 66 | 39,189 | 55 | 60 | 77 | |
| 72 | 51 | 58 | 41,580 | 65 | 66 | 77 | |
| 73 | 7 | 8 | 42,953 | 61 | 53 | 78 | |
| 74 | 11 | 16 | 43,146 | 70 | 57 | 76 | |
| 75 | 5 | 6 | 49,154 | 68 | 69 | 76 | |
| 76 | 5 | 11 | 57,641 | 75 | 74 | 78 | |
| 77 | 13 | 51 | 57,664 | 71 | 72 | 80 | |
| 78 | 5 | 7 | 64,635 | 76 | 73 | 79 | |
| 79 | 1 | 5 | 77,032 | 52 | 78 | 80 | |
| 80 | 1 | 13 | 163,220 | 79 | 77 | 0 | |


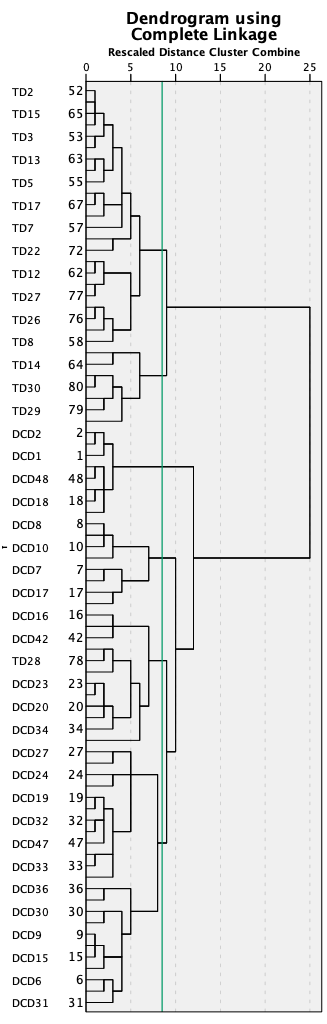


## Average linkage algorithm

| Agglomeration Schedule | | | | | | |  |
| --- | --- | --- | --- | --- | --- | --- | --- |
| Stage | Cluster Combined | | Coefficients | Stage Cluster First Appears | | Next Stage | |
|  | Cluster 1 | Cluster 2 |  | Cluster 1 | Cluster 2 |  |  |
| 1 | 52 | 56 | 4,084 | 0 | 0 | 5 | |
| 2 | 66 | 80 | 5,416 | 0 | 0 | 26 | |
| 3 | 74 | 76 | 6,002 | 0 | 0 | 33 | |
| 4 | 32 | 38 | 6,019 | 0 | 0 | 22 | |
| 5 | 52 | 65 | 6,229 | 1 | 0 | 12 | |
| 6 | 5 | 33 | 7,000 | 0 | 0 | 36 | |
| 7 | 19 | 44 | 7,326 | 0 | 0 | 30 | |
| 8 | 23 | 40 | 7,337 | 0 | 0 | 25 | |
| 9 | 22 | 48 | 7,382 | 0 | 0 | 21 | |
| 10 | 59 | 62 | 7,448 | 0 | 0 | 28 | |
| 11 | 9 | 21 | 7,720 | 0 | 0 | 17 | |
| 12 | 52 | 71 | 8,199 | 5 | 0 | 18 | |
| 13 | 2 | 43 | 8,235 | 0 | 0 | 35 | |
| 14 | 61 | 77 | 8,407 | 0 | 0 | 28 | |
| 15 | 3 | 18 | 8,602 | 0 | 0 | 21 | |
| 16 | 63 | 81 | 8,651 | 0 | 0 | 20 | |
| 17 | 9 | 15 | 9,379 | 11 | 0 | 40 | |
| 18 | 52 | 75 | 9,665 | 12 | 0 | 23 | |
| 19 | 54 | 67 | 9,866 | 0 | 0 | 39 | |
| 20 | 55 | 63 | 10,125 | 0 | 16 | 23 | |
| 21 | 3 | 22 | 10,306 | 15 | 9 | 34 | |
| 22 | 32 | 47 | 10,666 | 4 | 0 | 36 | |
| 23 | 52 | 55 | 10,863 | 18 | 20 | 43 | |
| ... |  |  |  |  |  |  | |
| 64 | 16 | 42 | 21,419 | 54 | 0 | 68 | |
| 65 | 27 | 41 | 22,809 | 0 | 0 | 77 | |
| 66 | 13 | 58 | 23,043 | 63 | 52 | 70 | |
| 67 | 7 | 17 | 24,357 | 53 | 60 | 71 | |
| 68 | 16 | 37 | 24,788 | 64 | 51 | 73 | |
| 69 | 5 | 6 | 24,899 | 62 | 59 | 74 | |
| 70 | 13 | 57 | 24,970 | 66 | 0 | 80 | |
| 71 | 7 | 34 | 25,278 | 67 | 0 | 73 | |
| 72 | 60 | 69 | 25,522 | 55 | 0 | 74 | |
| 73 | 7 | 16 | 27,851 | 71 | 68 | 76 | |
| 74 | 5 | 60 | 29,075 | 69 | 72 | 75 | |
| 75 | 5 | 24 | 30,021 | 74 | 61 | 77 | |
| 76 | 1 | 7 | 30,992 | 44 | 73 | 78 | |
| 77 | 5 | 27 | 33,552 | 75 | 65 | 78 | |
| 78 | 1 | 5 | 37,012 | 76 | 77 | 79 | |
| 79 | 1 | 11 | 43,395 | 78 | 0 | 80 | |
| 80 | 1 | 13 | 52,506 | 79 | 70 | 0 | |

**
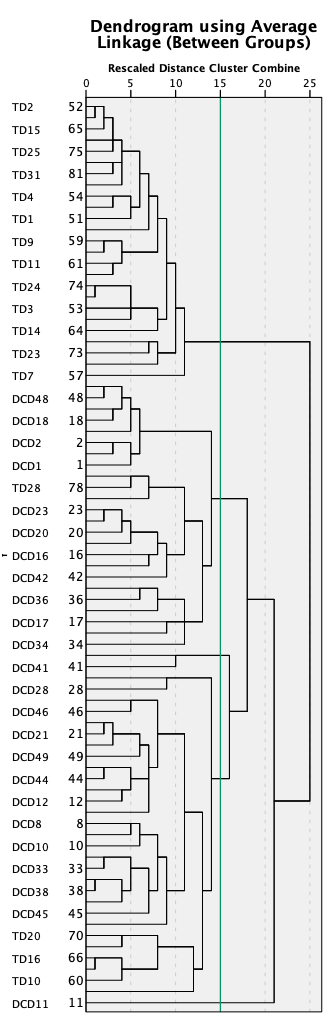
**
